# Lithium-Ion Battery Degradation: Measuring Rapid Loss of Active Silicon in Silicon–Graphite Composite Electrodes

**DOI:** 10.1021/acsaem.2c02047

**Published:** 2022-11-03

**Authors:** Niall Kirkaldy, Mohammad Amin Samieian, Gregory J. Offer, Monica Marinescu, Yatish Patel

**Affiliations:** † Department of Mechanical Engineering, Imperial College London, LondonSW7 2AZ, U.K.; ‡ The Faraday Institution, Harwell Science and Innovation Campus, DidcotOX11 0RA, U.K.

**Keywords:** lithium-ion batteries, aging, degradation modes, silicon, Si−Gr

## Abstract

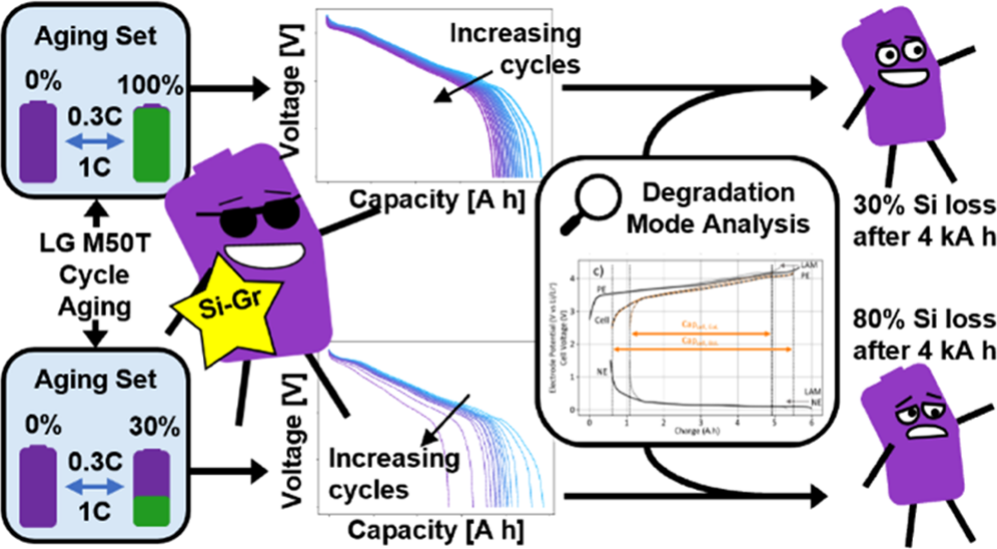

To increase the specific energy of commercial lithium-ion batteries, silicon is often blended into the graphite negative electrode. However, due to large volumetric expansion of silicon upon lithiation, these silicon–graphite (Si–Gr) composites are prone to faster rates of degradation than conventional graphite electrodes. Understanding the effect of this difference is key to controlling degradation and improving cell lifetimes. Here, the effects of state-of-charge and temperature on the aging of a commercial cylindrical cell with a Si–Gr electrode (LG M50T) are investigated. The use of degradation mode analysis enables quantification of separate rates of degradation for silicon and graphite and requires only simple in situ electrochemical data, removing the need for destructive cell teardown analyses. Loss of active silicon is shown to be worse than graphite under all operating conditions, especially at low state-of-charge and high temperature. Cycling the cell over 0–30% state-of-charge at 40 °C resulted in an 80% loss in silicon capacity after 4 kA h of charge throughput (∼400 equiv full cycles) compared to just a 10% loss in graphite capacity. The results indicate that the additional capacity conferred by silicon comes at the expense of reduced lifetime. Conversely, reducing the utilization of silicon by limiting the depth-of-discharge of cells containing Si–Gr will extend their lifetime. The degradation mode analysis methods described here provide valuable insight into the causes of cell aging by separately quantifying capacity loss for the two active materials in the composite electrode. These methods provide a suitable framework for any experimental investigations involving composite electrodes.

## Introduction

Enabled by their high energy density and specific energy, lithium-ion batteries (LIBs) have become the dominant energy storage technology for mobile applications. Average battery energy densities for electric vehicles (EVs) are rising at a rate of 7% per year.^1^ In the near term, they are expected to reach values of 325 W h kg^–1^ at the cell level, and 275 W h kg^–1^ at the pack level.^2^ This has been made possible using new active materials in their construction, such as nickel-rich transition metal oxides like LiNi_0.8_Mn_0.1_Co_0.1_O_2_ (NMC811) on the positive electrode (PE) and by incorporating silicon additives into the (typically) graphite negative electrode (NE).

Silicon is viewed as a promising NE material for LIBs due to its large specific capacity, which is around 10 times greater than that of graphite (3579 mA h g^–1^ for Li_15_Si_4_ vs 372 mA h g^–1^ for LiC_6_).^3^ Unlike intercalation materials such as graphite, silicon undergoes an alloying reaction upon lithiation. This leads to a significant volume expansion of over 300% in the fully lithiated state compared to graphite, which expands by around 20% upon lithiation.^4,5^

This expansion and contraction of the electrode during charge and discharge of the cell results in mechanical stress on the electrode particles.^6^ These stresses can cause particles to crack or even become completely detached from the rest of the electrode (known as island formation). These electrically isolated materials can no longer contribute to the capacity of the cell, hampering the performance.^7^ The large volumetric changes also cause the solid electrolyte interphase (SEI) layer to crack and become detached. This in turn leads to new electrode surfaces being exposed to the electrolyte, with additional SEI growth.^8^ Even under calendar aging conditions, SEI formation on Si is more dynamic and less passivating than that formed on graphite, leading to greater levels of degradation.^9,10^ Due to these issues, lifetimes of pure Si electrodes are often too short to be relevant for commercial applications.^11^

Some of the negative effects of Si-based electrodes can be mitigated through blending silicon or silicon oxides (SiO*_x_*) with other materials such as graphite. Silicon oxides undergo irreversible reduction reactions during the first lithiation cycle to form active silicon, inactive Li_2_O, and lithium silicates.^12−14^ Silicon–graphite (Si–Gr) composite electrodes have been shown to drastically improve cell lifetimes.^10,13^ Si–Gr electrodes with various structures and compositions have been explored to avoid the issues of electrical isolation and accelerated SEI growth.^15,16^

Due to the slightly higher oxidation potential of silicon vs graphite in a LIB, graphite is preferentially delithiated from a composite Si–Gr electrode during cell discharge. For an electrode containing relatively small amounts of silicon, the silicon portion of the composite Si–Gr electrode remains lithiated in all but the lowest states of charge (SoCs).^8,10^ This means that cycling a cell at high SoC will largely utilize the graphite content of the NE, whereas cycling at low SoCs will use a greater proportion of the Si content.^16^ Hence, the volumetric expansion of the silicon or graphite particles also depends on the SoC cycling range.^17^ We therefore hypothesize that the rate of cell level degradation will depend on the operational SoC window. We also expect that the relative aging rates of silicon and graphite in the NE vary with the SoC range.

Most degradation mechanisms in LIBs are in some way SoC (or potential)-dependent;^18^ mechanisms such as lithium-plating, SEI growth, and the various types of PE degradation are all exacerbated at higher cell SoC.^19−21^ Similarly, the majority of degradation mechanisms are dependent on cell temperature and current (or C-rate).^20^ Mechanisms that are caused by limited rates of Li^+^ diffusion through the electrolyte and active material particles are generally aggravated by operating the cell at low temperatures or high C-rates.^21^ This is due to slower transport at low temperatures and the formation of concentration gradients at high C-rates.^22,23^ Particle cracking is one such mechanism, since inhomogeneous lithiation will lead to greater strain on the active material particles and hence to greater rates of degradation.^23^

Identifying which degradation mechanisms have contributed to the performance drop seen during battery aging is a complex task. For verification of individual mechanisms, cell teardown must usually take place, with subsequent chemical, structural, and morphological analyses of the electrode and electrolyte.^7,24^ However, the consequences of different degradation mechanisms can be grouped together based on their impact on the electrochemical performance of the cell. These groupings are termed degradation modes (DMs), and usually consist of loss of active material (LAM), loss of lithium inventory (LLI), and resistance increase (RI).^25^ Each of these DMs can be quantified using in situ, nondestructive electrochemical methods without the need for the time-consuming, destructive ex situ methods required for identifying mechanisms directly.^26,27^

DM analyses can be performed directly on voltage vs capacity data^27−29^ or using a derivative such as incremental capacity (d*Q*/d*V*)^25,30,31^ or differential voltage (d*V*/d*Q*).^32−34^ Various studies have shown that these methods can be used to identify which DMs have contributed to cell-level performance drop, both qualitatively and quantitatively. The same methods have been applied to cells that contain composite electrodes, such as Si–Gr.^35,36^ Anseán et al. modeled the behavior of a Si–Gr/NMC cell during aging and showed that the LAM of the two components of the NE can be decoupled due to changes in the half-cell voltage curve.^36^ The profile of the NE voltage curve will change depending on the relative fractions of the two active materials, allowing insights into the way in which these composite electrodes age. This has since been confirmed experimentally through cell teardown and electrode harvesting.^37^ Schmitt et al. demonstrated that the composite electrode OCV-fitting method improved the validity of DM analysis when compared against methods that do not account for changes in the half-cell voltage curves.^38^

A range of commercial cells with Si–Gr negative electrodes have recently become available. Several experimental aging studies have sought to understand how these composite electrodes influence cell degradation and lifetime.^7,39−41^ These studies have provided valuable insight into the cell-level performance drop and the influence of various operating conditions. However, most have relied on destructive cell teardown analyses to disentangle the degradation contributions of the two components of the NE as the cell ages. Here, we seek to age commercial, Si–Gr-containing cells with relevance to the EV industry by cycling over different SoC windows and temperatures. We investigate how these conditions affect degradation through traditional methods of capacity fade, resistance increase, and incremental capacity analysis (ICA). We expand on this using the composite electrode OCV-fitting method to quantify which degradation modes occur during aging. This allows us to quantify not just LLI and LAM of the PE and NE but also LAM for both graphite and silicon. The resulting metrics provide greater insight into the relative merits of Si in these commercial cells, with an apparent trade-off between increased capacity and reduced lifetime.

## Experimental Section

In this study, we cycled commercial 21700 cylindrical cells (LG M50T, LG GBM50T2170) at different temperatures and SoC ranges. The LG M50T is a high-energy-density commercial cell with relevance to the EV industry. This cell utilizes a SiO*_x_*-doped graphite negative electrode alongside an NMC811 positive electrode and has a nominal capacity of 18.2 W h (5 A h).

Two sets of experiments were carried out, each seeking to age the cells in a different manner. The first set of experiments involved cycling the cell over the full SoC range (0–100%), i.e., full depth-of-discharge (DoD); this was expected to incite all types of cell degradation and acted as our “control” test. The second set of experiments restricted the SoC to the low region (0–30%), where we expected that the silicon portion of the NE would be more active than the graphite; we hypothesized that this would lead to greater rates of silicon degradation compared to the “control” test. Both sets of experiments were performed at three different temperatures: 10, 25, and 40 °C. The C-rates for charge (0.3C) and discharge (1C) were held constant throughout. A total of 17 cells were tested, distributed across the six experimental conditions (listed below). C-rates and capacities used for SoC control were based on the beginning-of-life (BoL) nominal capacity of 5 A h, i.e., 1C was equal to 5 A.

**Figure 1:**
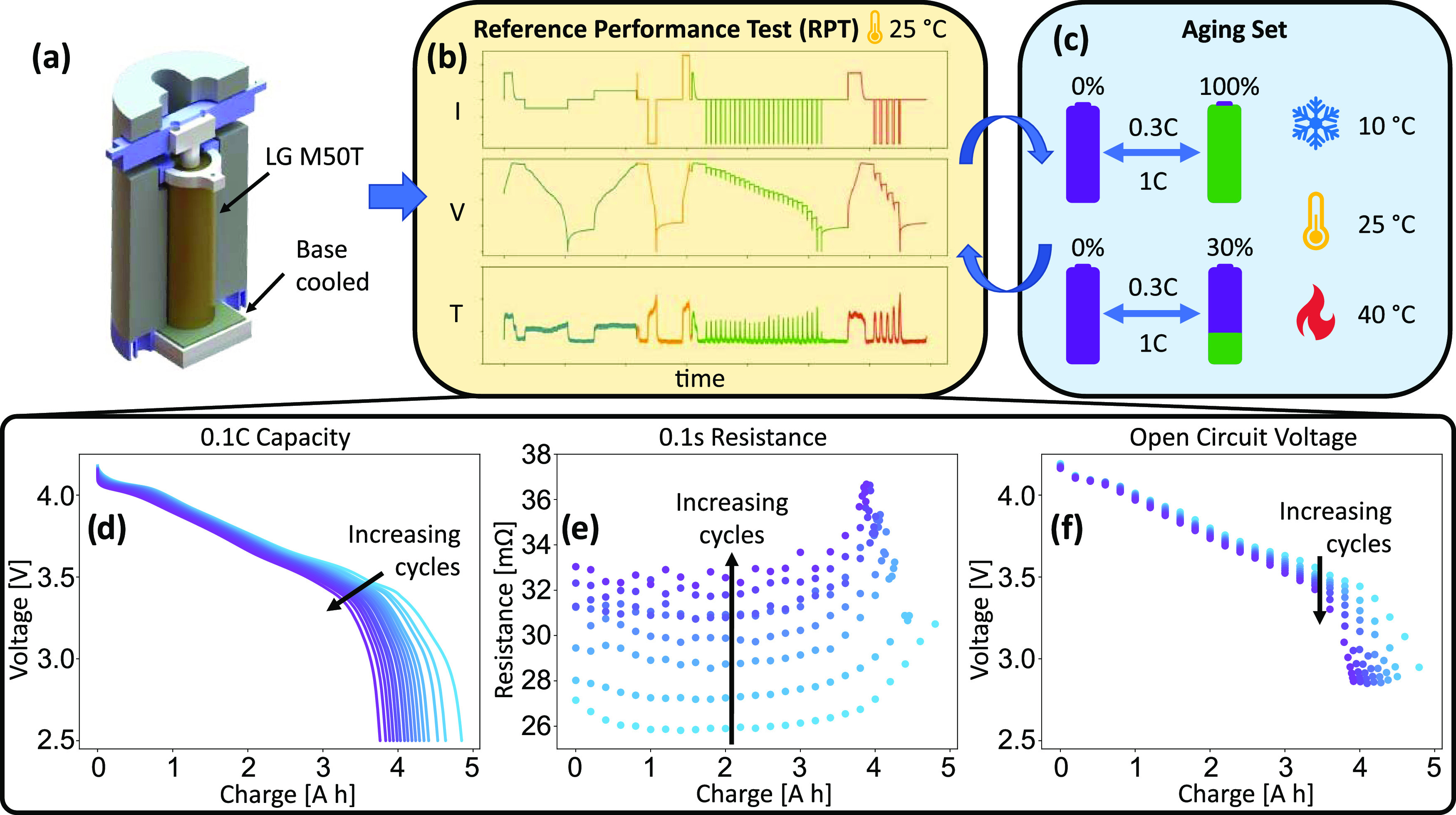
Schematic of the procedure used during this aging study. (a) Diagram of the test apparatus for a single cell. (b) Reference performance test (RPT) used at beginning-of-life and after each aging set. (c) Conditions used during cycle aging, with two SoC ranges and three temperature set-points. Cells were repeatedly subject to aging sets and RPTs until they were deemed to have reached end-of-life. Example data for a single cell extracted from the RPT procedure, showing (d) 0.1C discharge capacity, (e) 0.1-s resistance, and (f) open-circuit voltage.

Cells were thermally managed using bespoke test rigs (Figure 1a and SI Figures S1–S3). In these test rigs, the base (negative end) of each cylindrical cell was in thermal contact with an aluminum block which was held at a constant set-point temperature using Peltier elements. The rest of the cell was wrapped in thermal insulation to minimize heat loss through surfaces other than the base of the cell. This setup effectively sets a constant temperature boundary condition on the base of the cell, with pseudo-adiabatic conditions on the other surfaces, and is inspired by the thermal management strategy used in some EVs.^42^ Full details of the test rig design can be found in the Supporting Information (SI).

Once cells had been loaded into the test rigs, they were subject to five full discharge–charge cycles at a rate of 0.2C as part of the break-in procedure. The break-in procedure was necessary due to the empirical observation that the performance of commercial cells can change significantly over the first few cycles, often giving an apparent increase in capacity and reduction in resistance. These initial break-in cycles ensured that the cells were in a stable state at the beginning of the degradation study.

After the break-in procedure was complete, the BoL performance of the cells was measured using a reference performance test (RPT), detailed below and in Tables S1 and S2 of the SI. Two different RPT procedures were used in this study: the longer RPT provided more information on the cell performance but took around 100 h to complete; the shorter RPT provided less information but required around half the time. Both RPT procedures were performed at BoL and used alternatingly after each aging set. RPTs were always carried out at 25 °C, enabling comparisons between cells that were aged at different temperatures. The details of the longer RPT procedure are listed below and displayed in Figure 1b. Full details of both procedures can be found in Tables S1 and S2 of the SI.

The longer RPT can be broken down into four subtests, as indicated in the different colored portions of Figure 1b. This consisted of (i) a 0.1C discharge–charge (shown in blue in Figure 1b), (ii) a 0.5C discharge–charge (orange), and (iii/iv) two galvanostatic intermittent titration technique (GITT) discharge tests performed at 0.5C. The first of these (subtest iii) comprised of 25 pulses, each passing 200 mA h of charge, with 1-h rest periods between each pulse (green). The second (subtest iv) comprised of 5 pulses, each passing 1000 mA h of charge, also with 1-h rest periods between pulses (red). In all cases, voltage limits of 4.2 V (upper) and 2.5 V (lower) were imposed to prevent over-charge/discharge. Prior to each of the four subtests, the cells were first charged using a standard CC–CV method, with a C-rate of 0.3C until 4.2 V and a 4.2 V hold until the current dropped below 0.01C. The cells were then rested under open-circuit conditions for 2 h to allow the voltage and temperature to equilibrate. Upon reaching the 2.5 V lower voltage limit on discharge, cells were rested under open-circuit conditions for 6 h prior to commencing the next part of the procedure. The long rest periods were required due to the slow relaxation of these cells at low SoCs.

Once the BoL characterization was complete, the cells were brought to the temperature set-points required for their aging cycles and allowed to thermally equilibrate for 2 h. Two different SoC ranges were used in this study: 0–30 and 0–100% SoC. For each SoC range, three different temperature conditions were investigated: 10, 25, and 40 °C. Details of the cycling conditions are shown in Table 1 for the 0–30% SoC and Table 2 for the 0–100% SoC range.

**Table 1:** Cycling Conditions Used for Experiment 1 (0–30% SoC Cycling)

step	control type	control value	primary limits	end SoC	safety limits
1	CC discharge	1C	*E*_cell_ = 2.5 V	(0 + *x*)%	*E*_cell_ = 2.5 V
2	CV discharge	2.5 V	|*I*| < 0.01C	0%	N/A
3	rest	rest at OCV	time = 4 h	N/A	N/A
4	CC charge	0.3C	*Q* = 1500 mA h (=capacityBoL × 0.3)	30%	*E*_cell_ = 4.2 V
5	CC discharge	1C	*E*_cell_ = 2.5 V	0%	*E*_cell_ = 2.5 V
6	loop to step 4	N/A	257 times	N/A	N/A

**Table 2:** Cycling Conditions Used for Experiment 2 (Full Depth-of-Discharge)

step	control type	control value	primary limits	end SoC	safety limits
1	CC charge	0.3C	*E*_cell_ = 4.2 V	(100 – y)%	*E*_cell_ = 4.2 V
2	CV charge	4.2 V	|*I*| < 0.01C	100%	N/A
3	rest	rest at OCV	time = 4 h	N/A	N/A
4	CC discharge	1C	*E*_cell_ = 2.5 V	0%	*E*_cell_ = 2.5 V
5	CC charge	0.3C	*E*_cell_ = 4.2 V	(100 – y)%	*E*_cell_ = 4.2 V
6	CV charge	4.2 V	|*I*| < 0.01C	100%	N/A
7	loop to step 4	N/A	77 times	N/A	N/A

The aging sets described in Tables 1 and 2 consist of a set number of charge–discharge cycles. To maintain a fair comparison between the cells aged under different SoC windows, the number of cycles performed in each case was scaled to be equivalent in terms of number of full cycles. This meant that the full DoD (0–100% SoC) aging set had approximately 0.3 times the number of cycles of the 0–30% SoC aging set. After each set of aging cycles, cells were brought to 25 °C, and the performance of the cells was measured in another RPT. This process continued until the cells were deemed to have reached end-of-life (EoL).

The C-rates and capacities used for control of the SoC windows were not de-rated as the cells aged, with the nominal BoL capacities used throughout. A consequence of using coulomb-counting as the control method in the 0–30% SoC range cycling is that the voltage window over which the cell is cycled increases as the cell degrades, since the capacity used for setting the charge limit was not de-rated. Conversely, the cells cycled over the 0–100% SoC range are controlled by voltage set-points, meaning the charge passed during each aging set decreases as the cells degrade. These are unavoidable issues with aging studies of LIBs, but the impact on the results can be minimized by comparing against charge throughput rather than cycle number. Measurements of charge throughput were taken from the aging cycle data; this corresponds to the total measured charge passed during the aging cycles (from both charge and discharge sections).

Cell capacity measurements were taken from the 0.1C discharge cycle performed in each RPT (e.g., Figure 1d), from which “capacity fade” was calculated using the following equation:
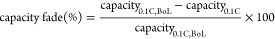
1

The resistance of the cells was calculated using the 25-pulse GITT data, from the instantaneous voltage drop upon application of the current pulse, as:
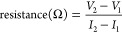
2where *I*_1_ and *V*_1_ are the current and voltage at rest before application of the current pulse and *I*_2_ and *V*_2_ are the values immediately after current is applied. The sampling rate for these measurements was 10 Hz, so the “instantaneous” voltage drop is approximated as that measured after 0.1 s. At this response time, some minor contributions from resistances other than ohmic (i.e., activation/charge transfer) are expected.

This procedure was repeated for each pulse in the GITT tests, giving resistance as a function of SoC (as shown in Figure 1e). The increase in resistance with aging was tracked for the resistance value thus obtained from the 12th pulse of the procedure (equating to ∼52% SoC using the nominal BoL capacity). This value was chosen due to the relative plateau in the BoL resistance values over the central SoC range of the cell. As with the capacity, the increase was defined relative to the BoL values (equation 3). It should be noted that for one cell aged in the 0–100% SoC range a faulty electrical connection during one of the GITT test resulted in erroneous results for RPT4; this datapoint has not been included in the subsequent analysis and the electrical connection was fixed prior to further cycling.
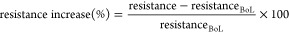
3Incremental capacity analysis (ICA) was performed using the 0.1C discharge and charge cycle by differentiating the cell capacity with respect to voltage using a finite-difference method with a set d*V* of 5 mV. Prior to this, the measured capacity was normalized using the nominal BoL capacity (5 A h) to give a measure of SoC. The differentiated data is therefore termed d*SoC*/d*V*. No smoothing was performed on the data.

Degradation mode (DM) analysis was performed through an OCV-fitting method using the 0.1C discharge data. OCV-fitting is an established method for quantifying degradation modes from full cell *V* vs *Q* data.^29^ A full cell OCV vs *Q* curve can be recreated from OCV vs *Q* datasets for the two individual electrodes (PE and NE). By scaling and shifting the *Q* values of the two electrodes, the calculated full cell *V* vs *Q* curve changes. By minimizing the difference between the calculated and experimentally measured full cell *V* vs *Q* curve, we can determine the capacities and offset of the two electrodes. Practically, this is achieved by adjusting the upper and lower lithiation fractions of each electrode (χ_PE_lo_, χ_PE_hi_, χ_NE_lo_, χ_NE_hi_) until the calculated *V* vs *Q* curve matches the measured data. This is done using a nonlinear least-squares fitting procedure, which minimizes the error between the calculated and measured full-cell voltage curves.

By repeating this procedure at different stages of degradation, we can track how the electrode capacities and offset change as the cell ages. Normalizing these against BoL values gives the degradation modes of LAM-PE, LAM-NE, and LLI, as discussed in the Introduction section.

The traditional OCV-fitting method relies on the *V* vs *Q* curves of the electrodes maintaining their shape, with only a uniform shrinkage/growth in the *x*-direction (*Q*) upon scaling. However, during aging, the shape of the composite electrode *V* vs *Q* curve can change due to the components aging at different rates. This ultimately means that it may not be possible to accurately recreate the *V* vs *Q* curve from a degraded cell using the BoL *V* vs *Q* curves of the two electrodes.

**Figure 2:**
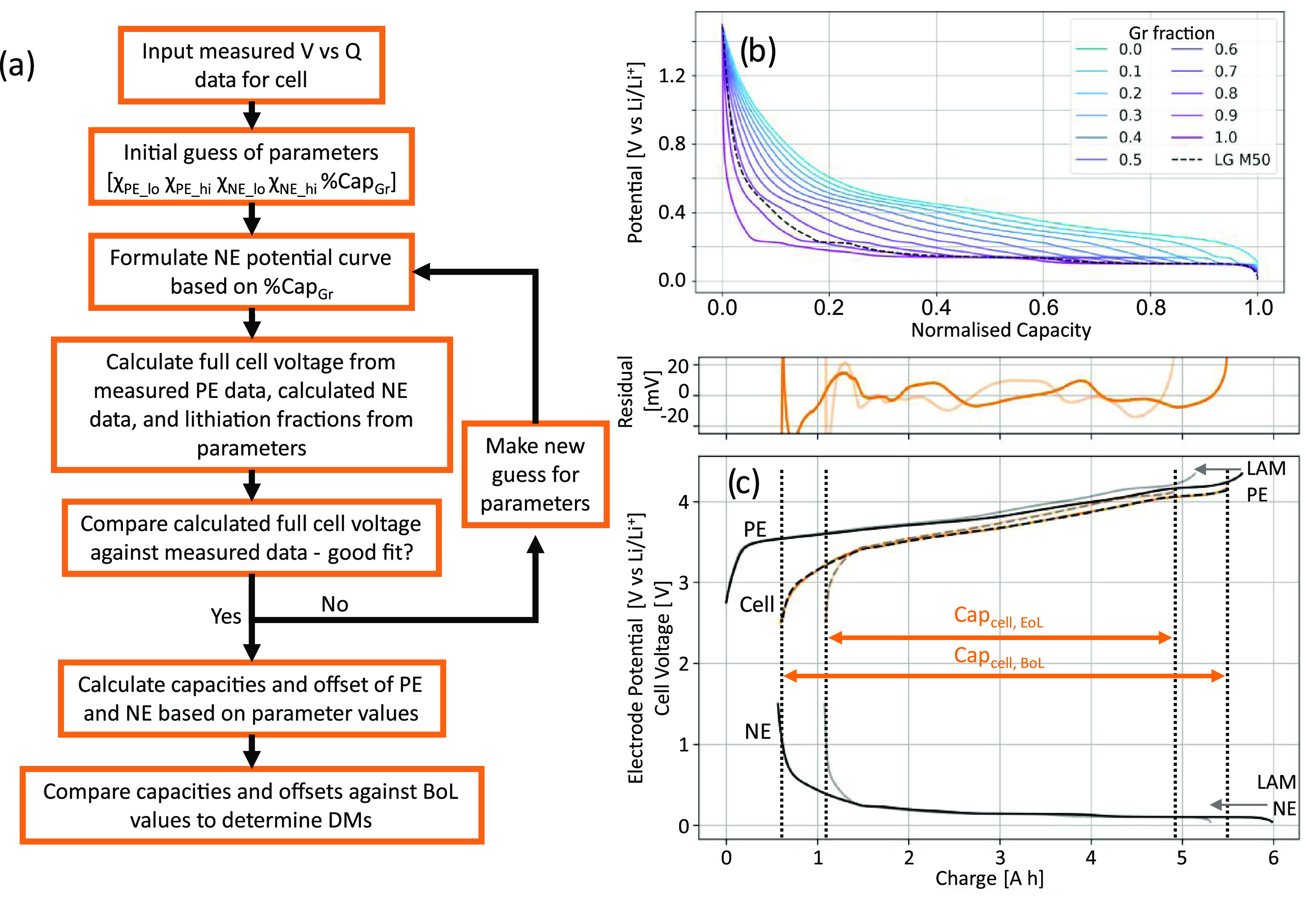
OCV-fitting procedure for quantifying the degradation modes. (a) Procedure for fitting the measured cell *V* vs *Q* data to a calculated *V* vs *Q* curve. (b) Calculated *V* vs (normalized) *Q* curves for different ratios of graphite and silicon in the composite NE alongside the experimentally measured data for the NE of the LG M50 cell (black dashed line). (c) Experimentally measured *V* vs *Q* curve of the PE is used together with the calculated NE curve from panel (b) to produce a full cell *V* vs *Q* curve based on the upper and lower lithiation fractions of each electrode. The plot shows measured (orange) and calculated (black dashed) full cell data at BoL (dark) and after aging (faded).

However, the OCV vs *Q* curve of a composite electrode is simply the sum of the two components (summing *Q* as a function of OCV, eq 4). OCV vs *Q* curves can therefore be calculated for different ratios of active material in the electrode (Figure 2b). For an electrode with two components, only one parameter (e.g., %Cap_Gr_) needs to be adjusted to account for these changing ratios, since the fraction of each component sums to one (eq 6).
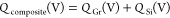
4
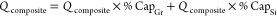
5
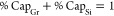
6It should be noted that eq 4 is valid only in the thermodynamic limit due to differing overpotentials present on the two components of the electrode. As such, it may only be suitable for low-current measurements (near equilibrium). This limitation can be overcome by introducing current-dependent half-cell data for each component.^36^

Introducing this additional parameter (%Cap_Gr_) into the OCV-fitting method enables the changing shape of the composite electrode curve to be modeled, which results in a more accurate fit of the full cell *V* vs *Q* curve. Importantly, it also provides a means of tracking the relative capacity contributions of each component of the composite electrode. This allows the calculation of the loss of each active material, LAM-NE_Gr_ and LAM-NE_Si_, instead of just the NE as a whole. This modified method therefore allows quantitative tracking of the rate of degradation of each component of the composite electrode, which cannot be achieved through the previous method, which utilizes static OCV curves for each electrode.

The OCV-fitting method described here requires half-cell *V* vs *Q* datasets for each component of the composite NE (pure Si and pure Gr), as well as the singular PE curve (NMC811). These were taken from the literature.^14,29,43^

We first investigated if the experimentally measured half-cell *V* vs *Q* curve for the Si–Gr composite NE could be recreated using the pure Si and Gr datasets. Adjusting the relative contributions of each component (i.e., %Cap_Gr_) alters the calculated *Q* vs *V* curve of the composite electrode (Figure 2b). A nonlinear least-squares fitting procedure was run using the Gr and Si datasets alongside a measured *V* vs *Q* curve of the NE at BoL (SI Figure S7). By minimizing the difference between the calculated and measured datasets, the optimum value of %Cap_Gr_ is found. From the experimentally measured *V* vs *Q* data for the NE of the LG M50 cell, the relative composition (in terms of capacity) was found to be 0.85 Gr: 0.15 Si at BoL (SI Figure S7).

Performing the full-cell OCV-fitting using the method described here gave a similar graphite-to-silicon capacity ratio of 0.87:0.13, thereby providing confidence in the ability to determine the composition of the negative electrode from full-cell analysis. More information on the fitting procedure and error of the fit can be found in the SI.

## Results and Discussion

**Figure 3:**
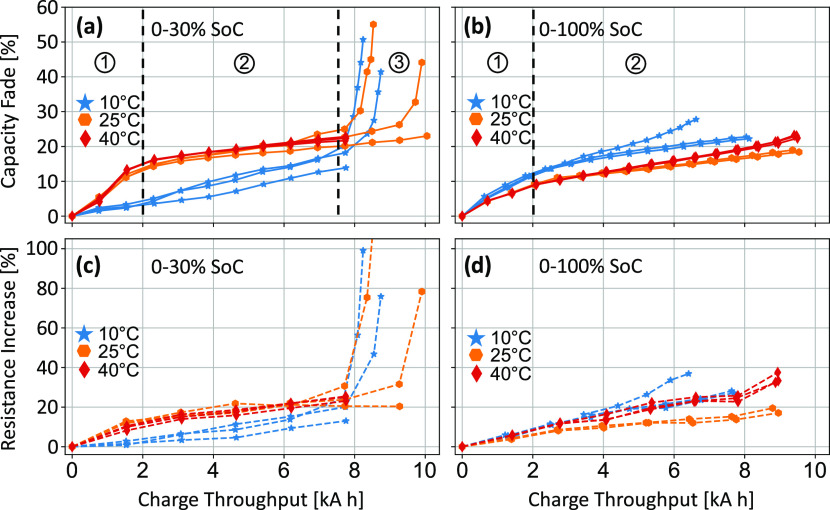
Capacity fade (a, b) and resistance increase (c, d) vs charge throughput for cells cycled at SoC ranges of 0–30% (a, c) and 0–100% (b,d). Colors correspond to the temperature at which the cells were aged (10 °C blue stars, 25 °C orange hexagons, and 40 °C red diamonds). Each line corresponds to an individual cell tested, showing the repeatability of cells aged under the same conditions. Capacities taken from 0.1C discharge at each point shown and resistances calculated from GITT measurements.

The various sections of the RPT procedure allow us to extract different pieces of information on the cells’ degradation behavior. From the 0.1C cycle, we can take a good estimate of the cell capacity. As described in eq 1, we can calculate the capacity fade as a function of charge throughput during the aging cycles. Figure 3a,b shows the capacity fade of each cell normalized against their BoL capacities.

The variance between cells was relatively low, with multiple repeats under each condition giving similar results (Figure 3a,b). The largest variance was observed when operating at a low temperature (10 °C), with lower variance seen at the mid (25 °C) and high (40 °C) temperatures. Overall, at 25°C and 40°C, lower rates of degradation were seen when operating over the 0–100% SoC range compared to the 0–30% SoC range. However, this is an incomplete story since different relationships are observed in the initial “accelerated aging” region and the latter “linear aging” region (indicated by regions 1 and 2 annotated in Figure 3). Low SoC cycling gives a greater initial capacity drop during the first 2 kA h of charge throughput before a slower linear capacity fade thereafter (region 2, Figure 3a). Conversely, full DoD cycling shows a smaller initial capacity drop, but the linear aging region progresses at a faster rate, evident from the steeper gradient (region 2, Figure 3b). In both the 0–30% and 0–100% SoC ranges, there is only a small difference between the observed capacity-fade behavior at 25°C and 40°C. However, there is a stark difference when operating at 10 °C. In the 0–100% SoC cycling, the cells at 10 °C age the fastest. The opposite behavior is seen in the cells cycled between 0–30% SoC, with the cells aged at 10 °C showing the lowest capacity fade for the first 7 kA h of charge throughput. As with the cells aged at warmer temperatures, the capacity-fade curve can be broken into two regions. The initial capacity fade seen during the “accelerated” stage (region 1) appears to be extremely low, but the rate of degradation seen in the “linear” region (region 2) is considerably higher than that seen at higher temperatures.

Region 3 of Figure 3a shows the “cliff edge” or “knee point” for some of the cells tested, where there is a sharp increase in capacity fade. This behavior has been observed in many experimental and modeling studies of lithium-ion battery aging.^44^ There are multiple possible causes of this behavior, which are the subject of ongoing research and are out-of-scope of this study.

The resistance increase follows a similar trend to the capacity fade discussed above (Figure 3c,d). It is therefore likely that the same degradation mechanisms which are causing capacity fade are also resulting in increased resistance. A decrease in capacity could result in an apparent increase in resistance. This is due to a decrease in the electrochemically active area of the electrode(s) while maintaining a constant resistivity. Conversely, a resistance increase can cause a reduction in the usable capacity of a cell due to premature activation of the voltage cutoff limits upon charge or discharge. However, since the capacity measurements were performed at a low rate of 0.1C, the effect of the resistance increase on the measured capacity is relatively small. At 0.1C (500 mA), an increase in resistance of 20% (∼5 mΩ) adds only 2.5 mV of overvoltage. The resistance measured here does not include overvoltages caused by an increase in the lower frequency dynamic (or “Faradaic”) resistances. However, due to the steep gradient of the *V* vs *Q* plots at low SoC, the amount of capacity “lost” through prematurely reaching the lower cutoff voltage is expected to be minimal.

Incremental capacity analysis (ICA) is a useful method for highlighting changes in cell voltage during aging. When performed on close-to-equilibrium data (i.e., low current), it can provide invaluable insight into the changes of the electrode compositions and capacities. Each peak in an ICA spectrum corresponds to a plateau in the voltage vs capacity (or SoC) profile.^25^ For full-cell data, a plateau on the voltage vs capacity profile arises due to an overlap between the plateaux of the constituent half-cell profiles. Hence, peaks on an ICA spectrum do not correspond to processes/features of any single electrode but instead to a combination of the features of the two electrodes.

**Figure 4:**
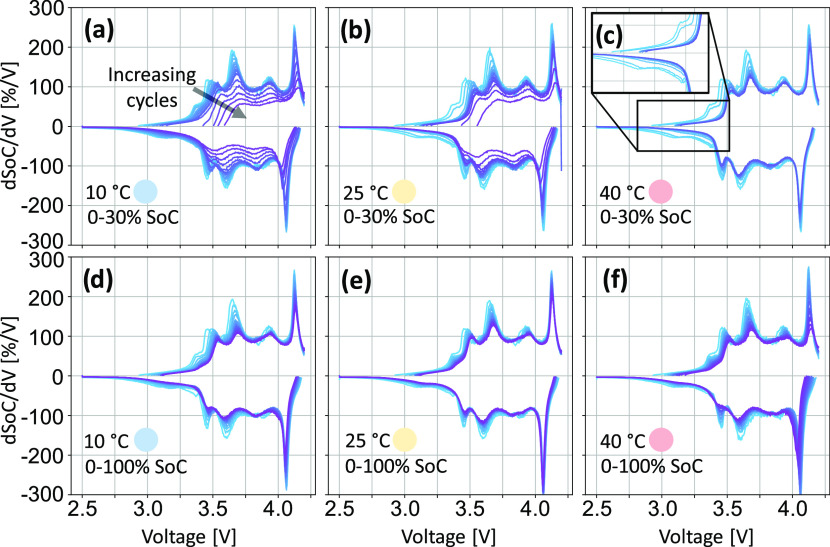
Progression of incremental capacity analysis (ICA) spectra throughout cell aging. Data from cells aged at 0–30% SoC (a–c) and 0–100% SoC (d–f), at temperatures of 10°C (a, d), 25 °C (b, e), and 40 °C (c, f). ICA calculated from the 0.1C discharge–charge cycle, with a fixed d*V* value of 5 mV. *Q* values were normalized by the nominal BoL capacity (5 A h) to give “SoC.” Different colors correspond to each RPT value, from BoL (light blue) to EoL (dark purple), with approximately 77 full equivalent cycles between each consecutive RPT.

Figure 4 shows the progression of the ICA spectra as the cells degrade, with data shown for one cell aged under each experimental condition. Figure 4a–c corresponds to cells cycled under the 0–30% SoC range, and Figure 4d–f corresponds to cells cycled under the 0–100% SoC range. The different colors within each plot correspond to a 0.1C (dis)charge cycle performed during each RPT, from BoL (light blue) to EoL (dark purple), with approximately 77 equiv full cycles between each consecutive RPT. Data for all cells can be found in SI Figures S8 and S9.

As discussed above, the peaks in an ICA plot cannot be attributed to one electrode. However, the peaks which correspond to (de)lithiation of silicon (alongside some PE feature) are principally observed in the low-voltage region (Figure S7). In particular, we attribute the broad, short negative peak between 2.7 and 3.3 V and the corresponding positive shoulder peak observed around 3.4 V to Si processes (inset, Figure 4c).^36^ It should be noted that the ICA spectrum of silicon is dependent on the voltage range over which it is cycled.^14^ These features rapidly disappear with increasing number of aging cycles, in particular, for the cells aged in the low SoC range (Figure 4a–c). As cells approach EoL, a dramatic collapse of all features in the ICA profiles can be observed, as demonstrated by the cell aged at 0–30% SoC and 10 °C (Figure 4a). The point at which this phenomenon is observed correlates to the “cliff edge” moment for capacity fade shown in Figure 3a (after ∼8 kA h of charge throughput). Further identification of the causes of degradation can be achieved through analysis of the degradation modes.

DM analysis of each cell was performed as outlined in the Experimental Section, using the 0.1C discharge data from the RPTs. The OCV-fitting procedure performed for each RPT allows us to calculate the capacities of the PE and NE and their relative offset, as well as the relative ratio of Gr:Si in the NE. By repeating the OCV-fitting at each stage of aging, we can track the evolution of the DMs as a function of charge throughput. This includes LLI and LAM of each electrode (LAM-PE and LAM-NE) as well as the LAM of each component of the NE (LAM-Gr and LAM-Si). It should be noted that this analysis cannot distinguish between loss of lithiated active material and loss of delithiated active material alongside LLI. The calculated LLI could therefore be caused by either stoichiometric drift (i.e., “electrode slippage”) due to mechanisms such as SEI formation or due to the LAM being lithiated material. Values of LLI have been normalized by dividing by the BoL cell capacity.

**Figure 5:**
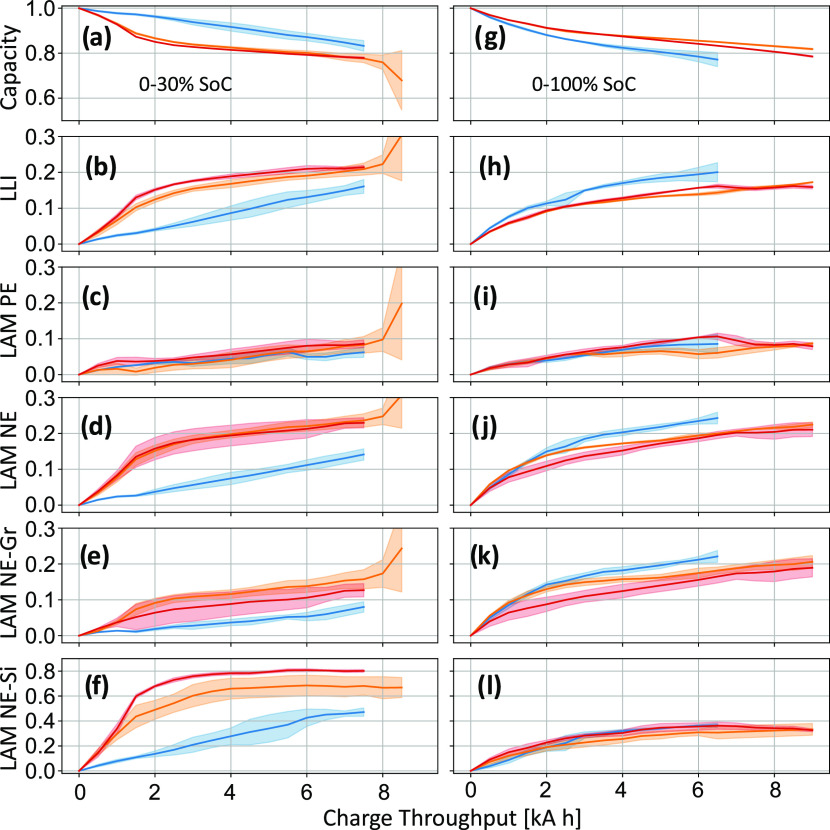
Degradation mode analysis for cells cycled at SoC ranges of 0–30% (a–f), and 0–100% (g–l). Colors correspond to cells aged at temperatures of 10°C (blue), 25 °C (orange), and 40 °C (red). Solid lines represent the average values calculated from multiple cells aged under the same condition, with shaded regions corresponding to the standard deviation. These plots show the evolution of normalized cell capacity and each degradation mode as a function of charge throughput during aging. LLI has been normalized by the BoL capacity of the cell.

The results from the DM analysis of cells aged by cycling within different SoC windows and temperatures are displayed in Figure 5. In these plots, multiple cells aged under the same conditions have been averaged to give the solid lines shown, with the shaded regions corresponding to the standard deviation. Results for each individual cell can be seen in SI Figures S11 and S13. In most cases, the root-mean-square error (RMSE) of the residual between the fitted OCV curve and the measured 0.1C data was found to be between 5 and 15 mV, with a gradual increase from BoL to EoL. This trend is expected due to the increasing resistance of the cells, causing the 0.1C discharge voltage to stray further away from the OCV.

Comparing the cells cycled over the 0–30% SoC range (Figure 5a–f) with those cycled between 0–100% SoC (Figure 5g–l) reveals significant differences in the DMs for the two operating ranges, in particular, for LAM-Si. For the cells aged at low SoC and mid/high temperature (Figure 5f), there is a greater than 70% loss in Si capacity after the first 4 kA h of energy throughput. This is expected, since the majority of the charge throughput in the 0–30% SoC range has been spent (de)lithiating silicon. Conversely, a greater proportion of the charge throughput in the 0–100% SoC range contributes toward (de)lithiating graphite, leading to higher levels of LAM-Gr in those cells (Figure 5k). LAM-Gr also rises in the 0–30% SoC cells once the silicon content is rendered inactive, since the graphite is now cycled exclusively (Figure 5e).

An appreciable amount of LAM-Si is also observed in the cells aged over the 0–100% SoC range, for which both the Si and Gr components are electrochemically cycled (note the different y-scales for LAM-Si and LAM-Gr on Figure 5). The high degree of LAM-Si relative to LAM-Gr may be caused by a variety of reasons: first, that the rate of Si degradation is inherently faster than that of Gr, in part due to the large volume changes experienced by Si upon (de)lithiation; second, due to the fact that the silicon particles experience far greater current densities than the graphite particles.^45^ This is an unavoidable problem faced by composite electrodes, resulting from the fact that the electrochemically active surface area of Si is significantly smaller than that of Gr for the material ratios usually used. Higher current densities lead to larger concentration gradients and increased rates of degradation. This effect worsens as LAM-Si increases, since the active surface area of silicon consequently decreases.

There is also a strong temperature dependence observed for the LAM-Si when cycling within the 0–30% SoC range (Figure 5f). Cells cycled at 10°C show lower levels of LAM-Si than those aged at 25 or 40 °C; they also have lower levels of LLI (Figure 5b). This may indicate that the increased rates of SEI growth expected at higher temperatures have a knock-on effect on the LAM, which is primarily caused by particle cracking. Thicker SEI layers can hinder diffusion processes, leading to larger concentration gradients and increased particle cracking. It is also possible that the low temperatures and resultant large overpotentials on the silicon active material cause the graphite to take on a larger portion of the charge throughput. These characteristics are expected to depend on the relative overpotentials of the two active materials, which depend on material properties and particle sizes and should be the focus of future work.

The temperature dependence of the LAM-Gr in the 0–100% SoC cells (Figure 5k) appears to be nonlinear; the highest levels of LAM-Gr are observed in the cells aged at 10 °C, while those aged at 25 and 40 °C show similar levels of material loss. This indicates that the kinetic limitations which cause high levels of particle cracking in Gr are abated by operating at temperatures of 25 °C and above.

Finally, similar levels of LAM-PE are observed in all cells tested here, regardless of the SoC range or temperature that the cells were cycled over (Figure 5c–i). This suggests that the degradation mechanisms responsible for the observed LAM-PE are not SoC-dependent. The relatively low levels of LAM-PE were expected due to the low-charging currents and adherence to the manufacturer’s upper voltage limits.

## Conclusions

We have investigated the effects of state-of-charge (SoC) and temperature on the degradation of commercial lithium-ion batteries with Si–Gr/NMC811 electrodes. Composite electrode open-circuit voltage modeling provided a means to separately quantify the capacities of graphite and silicon in the negative electrode and track the evolution of different degradation modes (DMs) during battery aging.

The results of the DM analysis performed in this work question the utility of silicon in these commercial cells. Silicon capacity loss of over 30% was observed after just 5 kA h of charge throughput, even when cycling under moderate conditions (0–100% SoC, 25 °C). For the same charge throughput, the loss in Si capacity was a staggering 80% when cycling over the 0–30% SoC range. These results indicate that the cycle life of cells containing Si–Gr NEs can be extended by reducing the amount of charge passed in the low SoC region. However, limiting the operational SoC window reduces the usable capacity of the cell, potentially removing the benefit of incorporating silicon into the electrode.

The degradation mode analytical method used here provides greater insight into the observed aging behavior by decoupling the LAM contributions from the two components of the NE. This methodology provides a suitable framework for the analysis of other experimental degradation studies on cells containing composite electrodes.

## Supporting Information Available

Additional experimental details, materials, and methods, including photographs of the experimental setup (PDF)

## Notes

Data supporting this study are openly available in Zenodo at https://doi.org/10.5281/zenodo.7235857.

## References

[ref1] Bloomberg NEF. Electric Vehicle Outlook. 2021.

[ref2] International Energy Agency. Innovation in Batteries and Electricity Storage. September, 2020, p 98.

[ref3] Obrovac M. N.; Christensen L. Structural Changes in Silicon Anodes during Lithium Insertion/Extraction. Electrochem. Solid-State Lett. 2004, 7, A93 10.1149/1.1652421.

[ref4] Beaulieu L. Y.; Hatchard T. D.; Bonakdarpour A.; Fleischauer M. D.; Dahn J. R. Reaction of Li with Alloy Thin Films Studied by In Situ AFM. J. Electrochem. Soc. 2003, 150, A1457 10.1149/1.1613668.

[ref5] Prado A. Y. R.; Rodrigues M.-T. F.; Trask S. E.; Shaw L.; Abraham D. P. Electrochemical Dilatometry of Si-Bearing Electrodes: Dimensional Changes and Experiment Design. J. Electrochem. Soc. 2020, 167, 160551 10.1149/1945-7111/abd465.

[ref6] Kumar R.; Tokranov A.; Sheldon B. W.; Xiao X.; Huang Z.; Li C.; Mueller T. In Situ and Operando Investigations of Failure Mechanisms of the Solid Electrolyte Interphase on Silicon Electrodes. ACS Energy Lett. 2016, 1, 689–697. 10.1021/acsenergylett.6b00284.

[ref7] Li X.; Colclasure A. M.; Finegan D. P.; Ren D.; Shi Y.; Feng X.; Cao L.; Yang Y.; Smith K. Degradation Mechanisms of High Capacity 18650 Cells Containing Si-Graphite Anode and Nickel-Rich NMC Cathode. Electrochim. Acta 2019, 297, 1109–1120. 10.1016/j.electacta.2018.11.194.

[ref8] Klett M.; Gilbert J. A.; Trask S. E.; Polzin B. J.; Jansen A. N.; Dees D. W.; Abraham D. P. Electrode Behavior RE-Visited: Monitoring Potential Windows, Capacity Loss, and Impedance Changes in Li 1.03 (Ni 0.5 Co 0.2 Mn 0.3) 0.97 O 2 /Silicon-Graphite Full Cells. J. Electrochem. Soc. 2016, 163, A875–A887. 10.1149/2.0271606jes.

[ref9] McBrayer J. D.; Rodrigues M. T. F.; Schulze M. C.; Abraham D. P.; Apblett C. A.; Bloom I.; Carroll G. M.; Colclasure A. M.; Fang C.; Harrison K. L.; Liu G.; Minteer S. D.; Neale N. R.; Veith G. M.; Johnson C. S.; Vaughey J. T.; Burrell A. K.; Cunningham B. Calendar Aging of Silicon-Containing Batteries. Nat. Energy 2021, 6, 866–872. 10.1038/s41560-021-00883-w.

[ref10] Eshetu G. G.; Zhang H.; Judez X.; Adenusi H.; Armand M.; Passerini S.; Figgemeier E. Production of High-Energy Li-Ion Batteries Comprising Silicon-Containing Anodes and Insertion-Type Cathodes. Nat. Commun. 2021, 12, 5459 10.1038/s41467-021-25334-8. 34526508 PMC8443554

[ref11] Cunningham B. Silicon and Intermetallic Anode Portfolio Strategy Overview. 2020.

[ref12] Kim T.; Park S.; Oh S. M. Solid-State NMR and Electrochemical Dilatometry Study on Li[Sup +] Uptake/Extraction Mechanism in SiO Electrode. J. Electrochem. Soc. 2007, 154, A1112 10.1149/1.2790282.

[ref13] Li H.; Li H.; Yang Z.; Yang L.; Gong J.; Liu Y.; Wang G.; Zheng Z.; Zhong B.; Song Y.; Zhong Y.; Wu Z.; Guo X. SiO _*x*_ Anode: From Fundamental Mechanism toward Industrial Application. Small 2021, 17, 2102641 10.1002/smll.202102641. 34553484

[ref14] Kitada K.; Pecher O.; Magusin P. C. M. M.; Groh M. F.; Weatherup R. S.; Grey C. P. Unraveling the Reaction Mechanisms of SiO Anodes for Li-Ion Batteries by Combining in Situ 7Li and Ex Situ 7Li/29Si Solid-State NMR Spectroscopy. J. Am. Chem. Soc. 2019, 141, 7014–7027. 10.1021/jacs.9b01589. 30964666

[ref15] Chae S.; Kim N.; Ma J.; Cho J.; Ko M. One-to-One Comparison of Graphite-Blended Negative Electrodes Using Silicon Nanolayer-Embedded Graphite versus Commercial Benchmarking Materials for High-Energy Lithium-Ion Batteries. Adv. Energy Mater. 2017, 7, 1700071 10.1002/aenm.201700071.

[ref16] Klett M.; Gilbert J. A.; Pupek K. Z.; Trask S. E.; Abraham D. P. Layered Oxide, Graphite and Silicon-Graphite Electrodes for Lithium-Ion Cells: Effect of Electrolyte Composition and Cycling Windows. J. Electrochem. Soc. 2017, 164, A6095–A6102. 10.1149/2.0131701jes.

[ref17] Yao K. P. C.; Okasinski J. S.; Kalaga K.; Almer J. D.; Abraham D. P. Operando Quantification of (De)Lithiation Behavior of Silicon–Graphite Blended Electrodes for Lithium-Ion Batteries. Adv. Energy Mater. 2019, 9, 1803380 10.1002/aenm.201803380.

[ref18] Edge J. S.; O’Kane S.; Prosser R.; Kirkaldy N. D.; Patel A. N.; Hales A.; Ghosh A.; Ai W.; Chen J.; Yang J.; Li S.; Pang M. C.; Bravo Diaz L.; Tomaszewska A.; Marzook M. W.; Radhakrishnan K. N.; Wang H.; Patel Y.; Wu B.; Offer G. J. Lithium Ion Battery Degradation: What You Need to Know. Phys. Chem. Chem. Phys. 2021, 23, 8200–8221. 10.1039/d1cp00359c. 33875989

[ref19] Lim J. M.; Hwang T.; Kim D.; Park M. S.; Cho K.; Cho M. Intrinsic Origins of Crack Generation in Ni-Rich LiNi0.8Co0.1Mn0.1O2 Layered Oxide Cathode Material. Sci. Rep. 2017, 7, 39669 10.1038/srep39669. 28045118 PMC5206713

[ref20] Jung R.; Strobl P.; Maglia F.; Stinner C.; Gasteiger H. A. Temperature Dependence of Oxygen Release from LiNi 0.6 Mn 0.2 Co 0.2 O 2 (NMC622) Cathode Materials for Li-Ion Batteries. J. Electrochem. Soc. 2018, 165, A2869–A2879. 10.1149/2.1261811jes.

[ref21] Waldmann T.; Wilka M.; Kasper M.; Fleischhammer M.; Wohlfahrt-Mehrens M. Temperature Dependent Ageing Mechanisms in Lithium-Ion Batteries - A Post-Mortem Study. J. Power Sources 2014, 262, 129–135. 10.1016/j.jpowsour.2014.03.112.

[ref22] Fan J.; Tan S. Studies on Charging Lithium-Ion Cells at Low Temperatures. J. Electrochem. Soc. 2006, 153, A1081 10.1149/1.2190029.

[ref23] Ai W.; Kraft L.; Sturm J.; Jossen A.; Wu B. Electrochemical Thermal-Mechanical Modelling of Stress Inhomogeneity in Lithium-Ion Pouch Cells. J. Electrochem. Soc. 2020, 167, 013512 10.1149/2.0122001jes.

[ref24] Henschel J.; Horsthemke F.; Stenzel Y. P.; Evertz M.; Girod S.; Lürenbaum C.; Kösters K.; Wiemers-Meyer S.; Winter M.; Nowak S. Lithium Ion Battery Electrolyte Degradation of Field-Tested Electric Vehicle Battery Cells – A Comprehensive Analytical Study. J. Power Sources 2020, 447, 227370 10.1016/j.jpowsour.2019.227370.

[ref25] Dubarry M.; Svoboda V.; Hwu R.; Liaw B. Y. Incremental Capacity Analysis and Close-to-Equilibrium OCV Measurements to Quantify Capacity Fade in Commercial Rechargeable Lithium Batteries. Electrochem. Solid-State Lett. 2006, 9, A454 10.1149/1.2221767.

[ref26] Dubarry M.; Liaw B. Y. Identify Capacity Fading Mechanism in a Commercial LiFePO4 Cell. J. Power Sources 2009, 194, 541–549. 10.1016/j.jpowsour.2009.05.036.

[ref27] Birkl C. R.; Roberts M. R.; McTurk E.; Bruce P. G.; Howey D. A. Degradation Diagnostics for Lithium Ion Cells. J. Power Sources 2017, 341, 373–386. 10.1016/j.jpowsour.2016.12.011.

[ref28] Birkl C. R.; McTurk E.; Roberts M. R.; Bruce P. G.; Howey D. A. A Parametric Open Circuit Voltage Model for Lithium Ion Batteries. J. Electrochem. Soc. 2015, 162, A2271–A2280. 10.1149/2.0331512jes.

[ref29] Prosser R.; Offer G.; Patel Y. Lithium-Ion Diagnostics: The First Quantitative In-Operando Technique for Diagnosing Lithium Ion Battery Degradation Modes under Load with Realistic Thermal Boundary Conditions. J. Electrochem. Soc. 2021, 168, 030532 10.1149/1945-7111/abed28.

[ref30] Dubarry M.; Truchot C.; Liaw B. Y.; Gering K.; Sazhin S.; Jamison D.; Michelbacher C. Evaluation of Commercial Lithium-Ion Cells Based on Composite Positive Electrode for Plug-in Hybrid Electric Vehicle Applications. Part II. Degradation Mechanism under 2 C Cycle Aging. J. Power Sources 2011, 196, 10336–10343. 10.1016/j.jpowsour.2011.08.078.

[ref31] Dubarry M.; Truchot C.; Liaw B. Y. Synthesize Battery Degradation Modes via a Diagnostic and Prognostic Model. J. Power Sources 2012, 219, 204–216. 10.1016/J.JPOWSOUR.2012.07.016.

[ref32] Bloom I.; Jansen A. N.; Abraham D. P.; Knuth J.; Jones S. A.; Battaglia V. S.; Henriksen G. L. Differential Voltage Analyses of High-Power, Lithium-Ion Cells 1. Technique and Application. J. Power Sources 2005, 139, 295–303. 10.1016/j.jpowsour.2004.07.021.

[ref33] Bloom I.; Christophersen J. P.; Abraham D. P.; Gering K. L. Differential Voltage Analyses of High-Power Lithium-Ion Cells: 3. Another Anode Phenomenon. J. Power Sources 2006, 157, 537–542. 10.1016/J.JPOWSOUR.2005.07.054.

[ref34] Bloom I.; Walker L. K.; Basco J. K.; Abraham D. P.; Christophersen J. P.; Ho C. D. Differential Voltage Analyses of High-Power Lithium-Ion Cells. 4. Cells Containing NMC. J. Power Sources 2010, 195, 877–882. 10.1016/j.jpowsour.2009.08.019.

[ref35] Ando K.; Matsuda T.; Imamura D. Degradation Diagnosis of Lithium-Ion Batteries with a LiNi0.5Co0.2Mn0.3O2 and LiMn2O4 Blended Cathode Using DV/DQ Curve Analysis. J. Power Sources 2018, 390, 278–285. 10.1016/j.jpowsour.2018.04.043.

[ref36] Anseán D.; Baure G.; González M.; Cameán I.; García A. B.; Dubarry M. Mechanistic Investigation of Silicon-Graphite/LiNi0.8Mn0.1Co0.1O2 Commercial Cells for Non-Intrusive Diagnosis and Prognosis. J. Power Sources 2020, 459, 227882 10.1016/j.jpowsour.2020.227882.

[ref37] Schmitt J.; Schindler M.; Jossen A. Change in the Half-Cell Open-Circuit Potential Curves of Silicon–Graphite and Nickel-Rich Lithium Nickel Manganese Cobalt Oxide during Cycle Aging. J. Power Sources 2021, 506, 230240 10.1016/j.jpowsour.2021.230240.

[ref38] Schmitt J.; Schindler M.; Oberbauer A.; Jossen A. Determination of Degradation Modes of Lithium-Ion Batteries Considering Aging-Induced Changes in the Half-Cell Open-Circuit Potential Curve of Silicon–Graphite. J. Power Sources 2022, 532, 231296 10.1016/j.jpowsour.2022.231296.

[ref39] Zilberman I.; Sturm J.; Jossen A. Reversible Self-Discharge and Calendar Aging of 18650 Nickel-Rich, Silicon-Graphite Lithium-Ion Cells. J. Power Sources 2019, 425, 217–226. 10.1016/j.jpowsour.2019.03.109.

[ref40] Wang C.; Amietszajew T.; Carvajal R.; Guo Y.; Ahmed Z.; Zhang C.; Goodlet G.; Bhagat R. Cold Ageing of NMC811 Lithium-Ion Batteries. Energies 2021, 14, 4724 10.3390/en14164724.

[ref41] Krause F. C.; Ruiz J. P.; Jones S. C.; Brandon E. J.; Darcy E. C.; Iannello C. J.; Bugga R. V. Performance of Commercial Li-Ion Cells for Future NASA Missions and Aerospace Applications. J. Electrochem. Soc. 2021, 168, 040504 10.1149/1945-7111/abf05f.

[ref42] Kyle W. B.; Tyler D. C.; Nathaniel C. W. Cylindrical Battery Cell Packaging and Cooling Configuration. US10886580B2, August 28, 2018.

[ref43] Chen C.-H.; Planella F. B.; O’Regan K.; Gastol D.; Widanage W. D.; Kendrick E. Development of Experimental Techniques for Parameterization of Multi-Scale Lithium-Ion Battery Models. J. Electrochem. Soc. 2020, 167, 80534 10.1149/1945-7111/ab9050.

[ref44] Attia P. M.; Bills A.; Brosa Planella F.; Dechent P.; dos Reis G.; Dubarry M.; Gasper P.; Gilchrist R.; Greenbank S.; Howey D.; Liu O.; Khoo E.; Preger Y.; Soni A.; Sripad S.; Stefanopoulou A. G.; Sulzer V. Review—“Knees” in Lithium-Ion Battery Aging Trajectories. J. Electrochem. Soc. 2022, 169, 060517 10.1149/1945-7111/ac6d13.

[ref45] Ai W.; Kirkaldy N.; Jiang Y.; Offer G.; Wang H.; Wu B. A Composite Electrode Model for Lithium-Ion Batteries with Silicon/Graphite Negative Electrodes. J. Power Sources 2022, 527, 231142 10.1016/j.jpowsour.2022.231142.

