# Supplementary material for: Lithium-Ion Battery Degradation: Measuring Rapid Loss of Active Silicon in Silicon–Graphite Composite Electrodes
Source: ACS Appl Energy Mater. 2022 Nov 3;5(11):13367–76. doi: 10.1021/acsaem.2c02047 (PMC9709825; doi:10.1021/acsaem.2c02047)
Supplement: Supplementary file 1 — ae2c02047_si_001.pdf [file ae2c02047_si_001.pdf]

Supporting Information

Lithium-ion battery degradation: measuring rapid loss of active silicon in silicon-graphite composite electrodes

Niall Kirkaldy [a], Mohammad Amin Samieian [a], Gregory J. Offer [a,b], Monica Marinescu [a,b], Yatish Patel* [a]*

[a] Department of Mechanical Engineering, Imperial College London, London, SW7 2AZ, United Kingdom.

[b] The Faraday Institution, Harwell Science and Innovation Campus, Didcot, OX11 0RA, United Kingdom

Corresponding Authors

Niall Kirkaldy – Department of Mechanical Engineering, Imperial College London, South Kensington Campus, London, United Kingdom; Email: n.kirkaldy@imperial.ac.uk

Yatish Patel – Department of Mechanical Engineering, Imperial College London, South Kensington Campus, London, United Kingdom; Email: yatish.patel@imperial.ac.uk1

Experimental Apparatus

Cells were thermally managed using bespoke test rigs, as shown in Figure S1, S2 & S3. In these test rigs, the base (negative end) of each cylindrical cell was in thermal contact with an aluminium block which was held at a constant set-point temperature. The rest of the cell was wrapped in thermal insulation (Ravatherm extruded polystyrene, thermal conductivity of 0.030 W m^{-1} K^{-1}) which had been machined to fit the dimensions of the cell. This mitigated heat loss through surfaces other than the base of the cell. The aluminium block at the base of the cell was held at a constant temperature using a PID controller on an Arduino Mega 2560. K-type thermocouples (TCs) provided the feedback for the control loop, which uses a Peltier element (20 W single-stage thermoelectric cooler, European Thermodynamics) to heat or cool the aluminium block based on the output current signal. Excess heat from the reverse side of the Peltier elements was extracted using an aluminium water-circulated cooling plate. The water was pumped through the cooling plate at a constant temperature using a CW-5200 industrial chiller. Thermal paste (10 W m^{-1} K^{-1}, Fischer Elektronik) or thermal interface material (12.5 W m^{-1} K^{-1}, t-Global Technology) were used at the interfaces between each component of the thermal apparatus to improve heat transfer. Cells were forced downwards onto the thermal interface block by the springs which held the electrical connections in place.

Surface temperature measurements of the cells were made using two K-type TCs per cell: one was recorded using a Pico TC-08 data logger (sampling frequency of 0.2 Hz), the other was recorded by the battery cycler whilst being used as its safety control. The TCs were adhered to the surface of the cell using Kapton tape, positioned halfway along the axial direction of the cell. The test rigs were found to be capable of holding the cells at stable temperatures of 5°C to 45°C, with fluctuations of ± 0.1°C while at rest.

Both the positive and negative electrical connections were made via the top of the cell. The negative connection was made via an aluminium, ring-shaped terminal which sat on the outer circumference of the top of the cell. The positive connection was made using an aluminium pin terminal which made contact with the positive cap. Both of these terminals were encased in plastic to prevent short-circuiting. The aluminium terminals were sanded and cleaned with isopropanol prior to use. Cell terminals were also cleaned with isopropanol. The connections were forced down into the top of the cells using springs which were anchored to the cooling plate at the base of the cells.

The terminals were connected to the battery cycler (Biologic BCS-815) current and voltage-sense cables via banana plugs. Cell impedance was tested after set-up by using the iR-compensation function of the BCS-815 at a frequency of 10 kHz and amplitude of 5 mV, averaging over 4 measurements. The total impedance (including ohmic resistance from the cell itself) was consistently found to be 27-30 mΩ; the cell itself was found to be 27 mΩ when measured using a 4-point connection, indicating the electrical connections added < 3 mΩ. The resistance was not compensated for in subsequent tests.

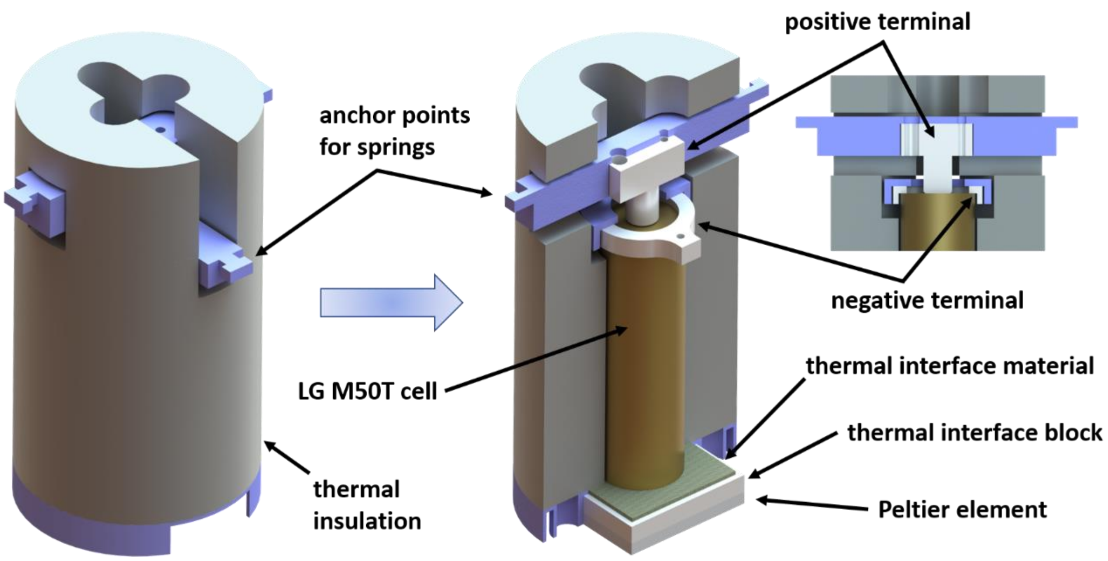

Figure S1. Schematic of the test rig used in this study. The cell sits atop an aluminium block which is held at a constant temperature using a Peltier element connected to a PID controller. The other surfaces of the cell are encased in thermal insulation which had been CNC machined to the dimensions of the cell. Electrical connections are via ring and pin terminals at the top of the cell. A water-cooled plate (not shown) removes excess heat from the opposite side of the Peltier elements.

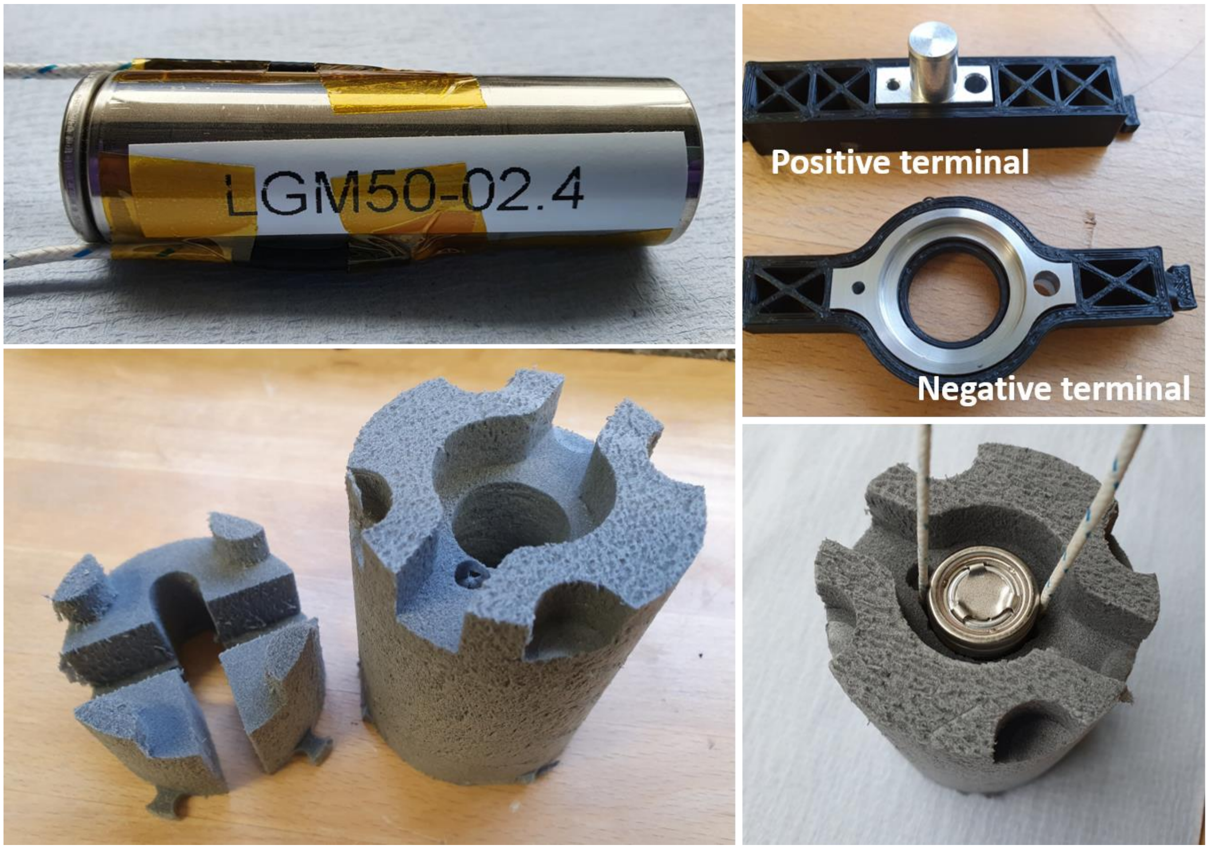

Figure S2. Photographs showing (clockwise from top left): an LG M50T cell used in this study, with two thermocouples adhered to the surface; the positive and negative electrical terminals for connecting to the cell; a cell with two TCs attached to the surface, sitting inside the thermal insulation; the body (right) and lid (left) of the thermal insulation.

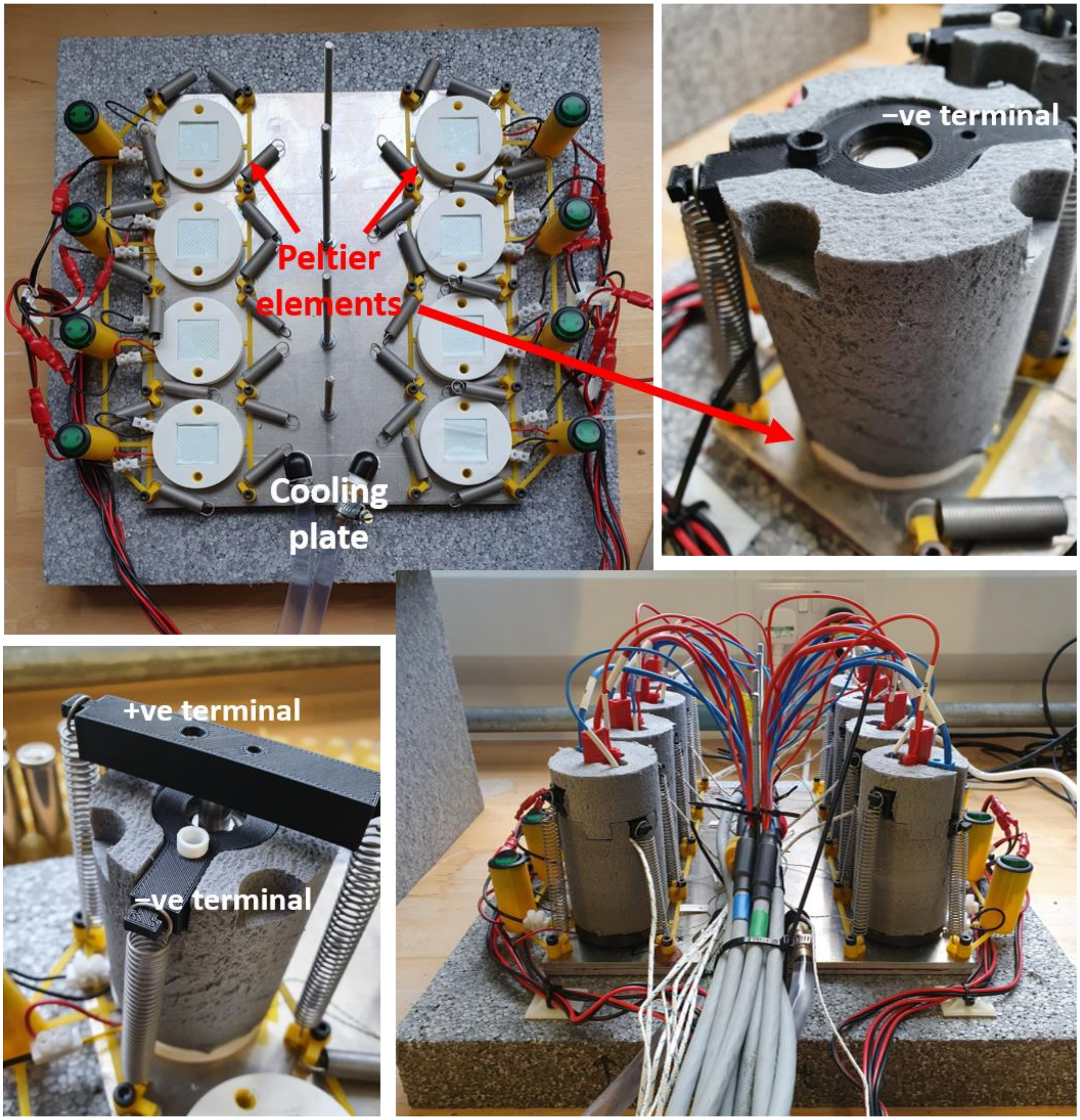

Figure S3. Photographs showing (clockwise from top left): a rig which can independently thermally manage 8 cells, each with their own Peltier element/interface block, sitting on top of a water-cooled plate; a cell wrapped in insulation sitting on one of the interface blocks, with springs holding the negative electrode ring terminal in place; 8 cells loaded onto the test rig, each with independent temperature, voltage and current control; a cell wrapped in insulation sitting on one

of the interface blocks, with springs holding the negative electrode ring terminal and positive electrode pin terminal in place.

Break-In Cycles

The break-in cycles consisted of cycling cells between the upper and lower voltage limits (4.2 V and 2.5 V) using the standard charge and discharge procedures outlined in the manufacturer's specification sheet. These were a constant-current, constant-voltage (CC-CV) charge (with a C-rate of 0.2C until 4.2 V, and a 4.2 V hold until the current dropped below 0.01C), and a CC discharge (with a C-rate of 0.2C). Cells were rested under open-circuit conditions for 2 hours after each charge and 4 hours after each discharge. All break-in cycles were performed at 25°C.

Reference Performance Test (RPT)

As a balance between gaining important information on the performance of the cell and not expending too much time (and charge) outside of the aging sets, two different RPT procedures were used in this study. The longer procedure was performed after each even-numbered aging set, whereas the shorter procedure was used after each odd-numbered aging set. Both procedures were run at BoL after the break-in cycles had been performed. RPTs were always performed at 25°C. Once the RPT had finished, prior to the next aging set commencing, cells were returned to approximately 50% SoC by charging at 0.3C until the cell voltage reached 3.7 V. The two procedures are detailed in Table S1 & S2, and shown visually for an example cell in Figure S4 & S5.

Table S1. The long version of the RPT procedure, run on every cell after each even-numbered aging set.

Sub-Test	Step	Control Type	Control Value	Primary Limits	Safety Limits
1. C/10 CC	1.1	CC charge	0.3C	E_{cell} = 4.2 V	E_{cell} = 4.2 V
	1.2	CV charge	4.2 V	|I| < 0.01C	N/A
	1.3	Rest	Rest at OCV	time = 2 hours	N/A
	1.4	CC discharge	0.1C	E_{cell} = 2.5 V	E_{cell} = 2.5 V
	1.5	Rest	Rest at OCV	time = 6 hours	N/A
	1.6	CC charge	0.1C	E_{cell} = 4.2 V	E_{cell} = 4.2 V
	1.7	Rest	Rest at OCV	time = 10 mins	N/A
2. C/2 CC	2.1	CC charge	0.3C	E_{cell} = 4.2 V	E_{cell} = 4.2 V
	2.2	CV charge	4.2 V	|I| < 0.01C	N/A
	2.3	Rest	Rest at OCV	time = 2 hours	N/A
	2.4	CC discharge	0.5C	E_{cell} = 2.5 V	E_{cell} = 2.5 V
	2.5	Rest	Rest at OCV	time = 6 hours	N/A
	2.6	CC charge	0.5C	E_{cell} = 4.2 V	E_{cell} = 4.2 V
	2.7	Rest	Rest at OCV	time = 30 mins	N/A
3. C/2 GITT (25 pulses)	3.1	CC charge	0.3C	E_{cell} = 4.2 V	E_{cell} = 4.2 V
	3.2	CV charge	4.2 V	|I| < 0.01C	N/A
	3.3	Rest	Rest at OCV	time = 2 hours	N/A
	3.4	CC discharge	0.5C	Q = 200 mA h	E_{cell} = 2.5 V
	3.5	Rest	Rest at OCV	time = 1 hour	N/A
	3.6	Loop to step 3.4	N/A	24 times	N/A
	3.7	Rest	Rest at OCV	time = 5 hours	N/A
4. C/2 GITT (5 pulses)	4.1	CC charge	0.3C	E_{cell} = 4.2 V	E_{cell} = 4.2 V
	4.2	CV charge	4.2 V	|I| < 0.01C	N/A
	4.3	Rest	Rest at OCV	time = 2 hours	N/A
	4.4	CC discharge	0.5C	Q = 1000 mA h	E_{cell} = 2.5 V
	4.5	Rest	Rest at OCV	time = 1 hour	N/A
	4.6	Loop to step 4.4	N/A	4 times	N/A
	4.7	Rest	Rest at OCV	time = 5 hours	N/A

Table S2. The short version of the RPT procedure, run on every cell after each odd-numbered aging set.

Sub-Test	Step	Control Type	Control Value	Primary Limits	Safety Limits
1. C/10 CC	1.1	CC charge	0.3C	E_{cell} = 4.2 V	E_{cell} = 4.2 V
	1.2	CV charge	4.2 V	|I| < 0.01C	N/A
	1.3	Rest	Rest at OCV	time = 2 hours	N/A
	1.4	CC discharge	0.1C	E_{cell} = 2.5 V	E_{cell} = 2.5 V
	1.5	Rest	Rest at OCV	time = 6 hours	N/A
	1.6	CC charge	0.1C	E_{cell} = 4.2 V	E_{cell} = 4.2 V
	1.7	Rest	Rest at OCV	time = 10 mins	N/A
2. Pulse under load (C/2 average current, discharge and charge)	2.1	CC charge	0.3C	E_{cell} = 4.2 V	E_{cell} = 4.2 V
	2.2	CV charge	4.2 V	|I| < 0.01C	N/A
	2.3	Rest	Rest at OCV	time = 2 hours	N/A
	2.4	CC discharge	0.5C	t = 30 s	E_{cell} = 2.5 V
	2.5	CC discharge	0.55C	t = 10 s	E_{cell} = 2.5 V
	2.6	CC discharge	0.5C	t = 30 s	E_{cell} = 2.5 V
	2.7	CC discharge	0.45C	t = 10 s	E_{cell} = 2.5 V
	2.8	CC discharge	0.5C	t = 30 s	E_{cell} = 2.5 V
	2.9	CC discharge	0.625C	t = 10 s	E_{cell} = 2.5 V
	2.10	CC discharge	0.5C	t = 30 s	E_{cell} = 2.5 V
	2.11	CC discharge	0.375C	t = 10 s	E_{cell} = 2.5 V
	2.12	CC discharge	0.5C	t = 30 s	E_{cell} = 2.5 V
	2.13	CC discharge	0.75C	t = 10 s	E_{cell} = 2.5 V
	2.14	CC discharge	0.5C	t = 30 s	E_{cell} = 2.5 V
	2.15	CC discharge	0.25C	t = 10 s	E_{cell} = 2.5 V
	2.16	Loop to step 2.4	N/A	29 times	N/A
	2.17	Rest	Rest at OCV	time = 6 hours	N/A
	2.18	CC charge	0.5C	t = 30 s	E_{cell} = 4.2 V
	2.19	CC charge	0.55C	t = 10 s	E_{cell} = 4.2 V
	2.20	CC charge	0.5C	t = 30 s	E_{cell} = 4.2 V
	2.21	CC charge	0.45C	t = 10 s	E_{cell} = 4.2 V
	2.22	CC charge	0.5C	t = 30 s	E_{cell} = 4.2 V

	2.23	CC charge	0.625C	t = 10 s	E_{cell} = 4.2 V
	2.24	CC charge	0.5C	t = 30 s	E_{cell} = 4.2 V
	2.25	CC charge	0.375C	t = 10 s	E_{cell} = 4.2 V
	2.26	CC charge	0.5C	t = 30 s	E_{cell} = 4.2 V
	2.27	CC charge	0.75C	t = 10 s	E_{cell} = 4.2 V
	2.28	CC charge	0.5C	t = 30 s	E_{cell} = 4.2 V
	2.29	CC charge	0.25C	t = 10 s	E_{cell} = 4.2 V
	2.30	Loop to step 2.18	N/A	29 times	N/A
	2.31	Rest	Rest at OCV	time = 10 mins	N/A
3. Pulse under load (1C average current, discharge only)	3.1	CC charge	0.3C	E_{cell} = 4.2 V	E_{cell} = 4.2 V
	3.2	CV charge	4.2 V	|I| < 0.01C	N/A
	3.3	Rest	Rest at OCV	time = 2 hours	N/A
	3.4	CC discharge	1C	t = 30 s	E_{cell} = 2.5 V
	3.5	CC discharge	1.1C	t = 10 s	E_{cell} = 2.5 V
	3.6	CC discharge	1C	t = 30 s	E_{cell} = 2.5 V
	3.7	CC discharge	0.9C	t = 10 s	E_{cell} = 2.5 V
	3.8	CC discharge	1C	t = 30 s	E_{cell} = 2.5 V
	3.9	CC discharge	1.25C	t = 10 s	E_{cell} = 2.5 V
	3.10	CC discharge	1C	t = 30 s	E_{cell} = 2.5 V
	3.11	CC discharge	0.75C	t = 10 s	E_{cell} = 2.5 V
	3.12	CC discharge	1C	t = 30 s	E_{cell} = 2.5 V
	3.13	CC discharge	1.5C	t = 10 s	E_{cell} = 2.5 V
	3.14	CC discharge	1C	t = 30 s	E_{cell} = 2.5 V
	3.15	CC discharge	0.5C	t = 10 s	E_{cell} = 2.5 V
	3.16	Loop to step 3.4	N/A	14 times	N/A
	3.17	Rest	Rest at OCV	time = 6 hours	N/A

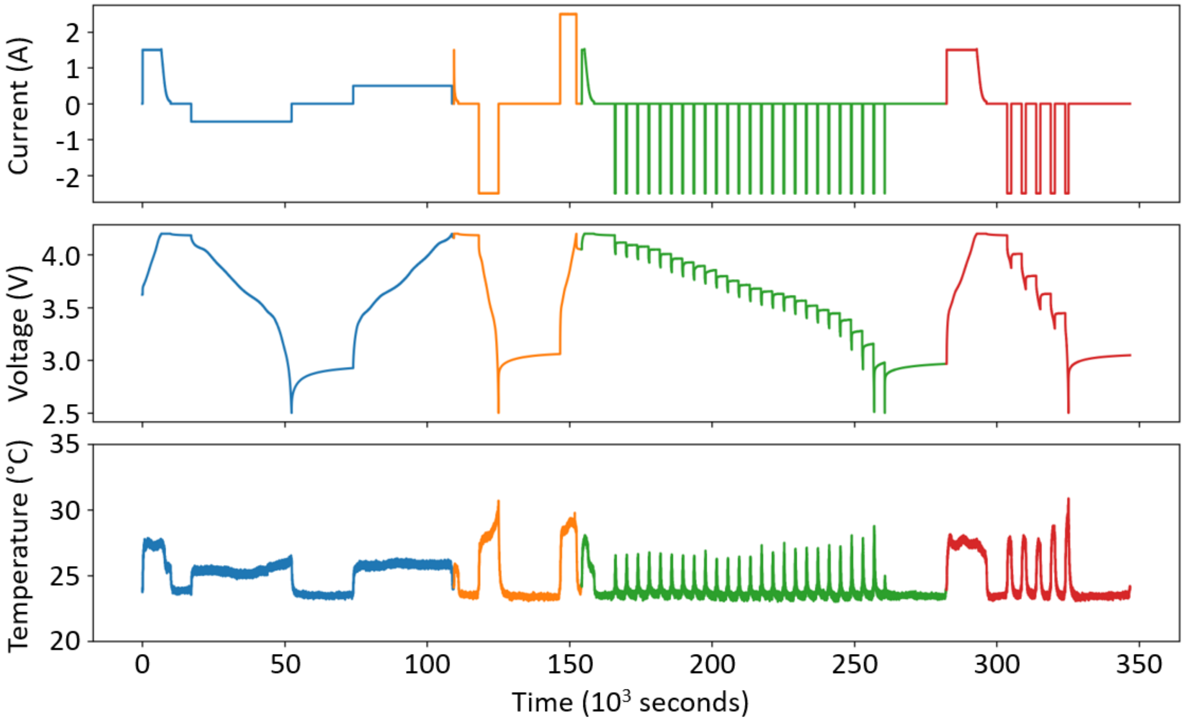

Figure S4. The longer version of the RPT procedure, showing current (top), voltage (mid), and temperature (bottom) versus time. This procedure was performed on every cell after each even-numbered aging set. Colors correspond to the various sub-tests described in Table S1.

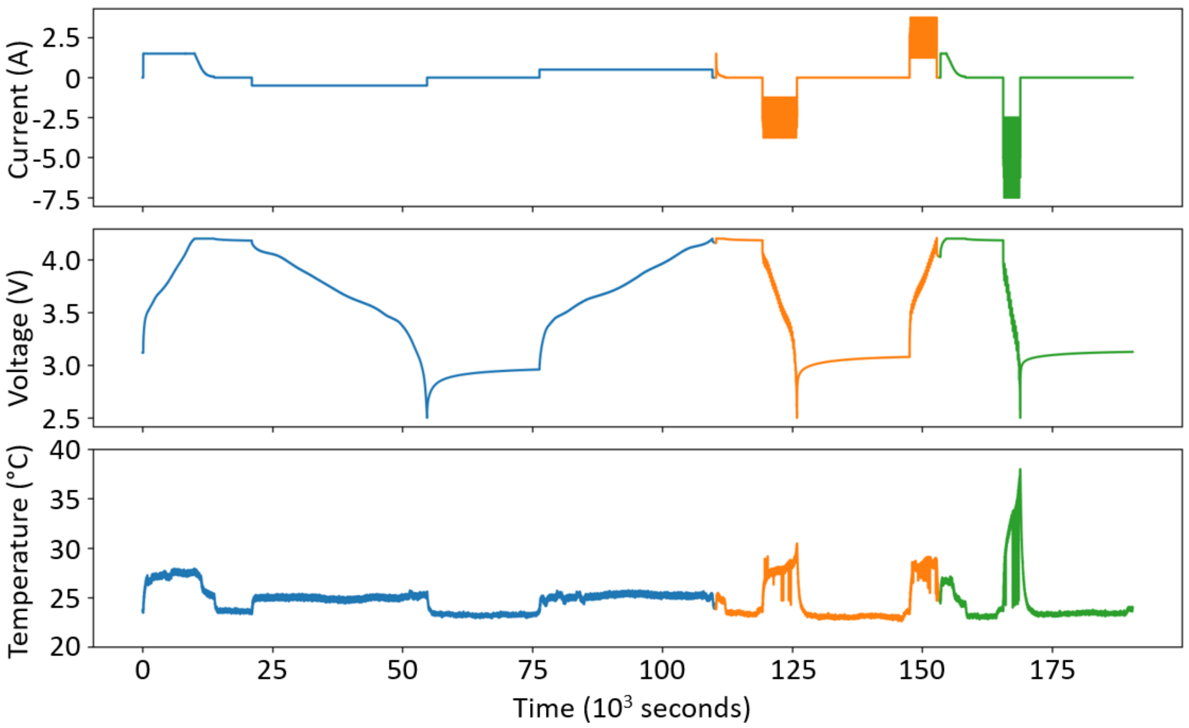

Figure S5. The shorter version of the RPT procedure, showing current (top), voltage (mid), and temperature (bottom) versus time. This procedure was performed on every cell after each odd-numbered aging set. Colors correspond to the various sub-tests described in Table S2.

Data Analysis

All analyses of the electrochemical data were performed using Python, making use of the Pandas, Numpy, Scipy, and Matplotlib libraries. Measurements of charge throughput were taken from the aging cycle data; this corresponds to the total measured charge passed during the aging cycles (from both charge and discharge sections). Aside from the measure of charge throughput, all other information used in the rest of the analyses utilize data from the RPTs, as described in the main text.

Cell-to-Cell Variation

To compare the variability between cells at BoL, the 0.1C discharge capacities for 40 cells were compared. These data were recorded on cells which had undergone break-in cycles before being tested as per the 'long' RPT procedure described above. This revealed an average capacity of 4864.91 mA h, with a standard deviation of 20.52 mA h (0.42% of mean).

Aging Cycles

The conditions used in the aging cycles are described in Table 1 and 2 of the main text. Table S3 below summarises the different conditions and the number of cells tested under each. Figure S6 shows the current and voltage profiles for some example aging cycles for the two SoC ranges used in this study.

Table S3. Experimental conditions used in the aging study. Cells were cycled over two SOC regions and three temperatures, giving six different conditions. Multiple cells were cycled under each condition. The charge (C) and discharge (D) C-rates were the same in all cases.

Experiment	SOC Window	Current	Temperature	Number of Cells
1	0-30%	0.3C / 1D	10°C	3
			25°C	3
			40°C	3
2	0-100%	0.3C / 1D	10°C	3
			25°C	2
			40°C	3

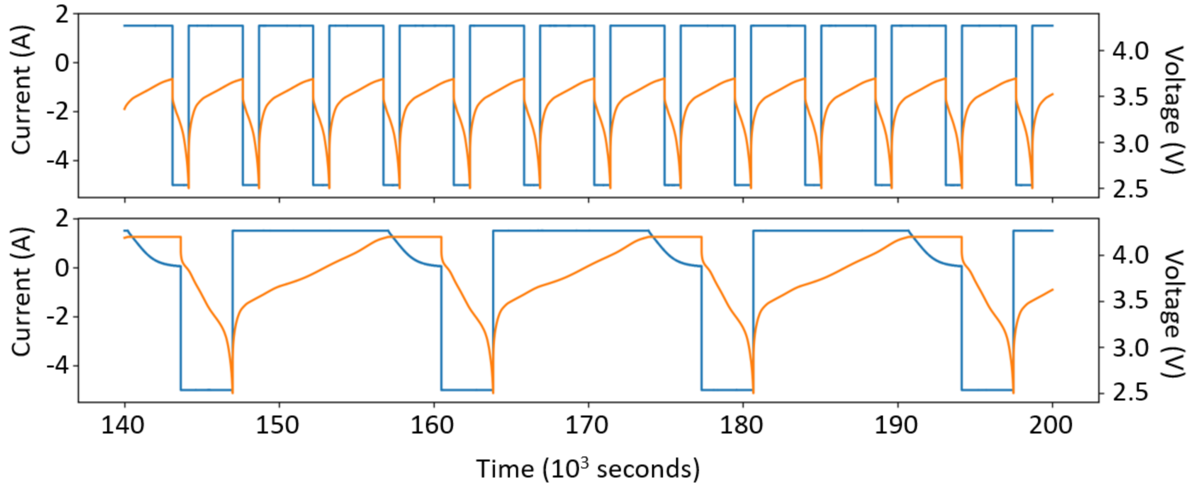

Figure S6. Example of some aging cycles for the two SoC ranges used in this study, with 0-30% SoC cycling (top) and 0-100% SoC cycling (bottom). Shown is the current (blue, left axis) and voltage (orange, right axis) vs time for a short time period during one of the aging sets. The total number of full charge-discharge cycles per aging set were 77 for the 0-100% SoC range, and 257 for the 0-30% cells (≈77/0.3).

DM Analysis

To calculate the fractional capacity of graphite (Gr) and silicon in the negative electrode (NE), we compared the measured Si-Gr voltage (V) vs capacity (Q) delithiation profile for the NE (taken from reference [1]) against separate measured V vs Q profiles for Si and Gr delithiation (refs. [2] and [3], respectively). As described in the main text, the capacity of a composite electrode at any given V is the sum of the constituent parts. By altering the Gr:Si ratio for the calculated Q vs V curve, we can obtain a fit for the experimentally measured Q vs V curve for the Si-Gr NE (Figure S7). This was done using the curve_fit function of the SciPy library in Python, using a Trust Region Reflective algorithm with bounds of [0, 1] for the fraction of Gr. The resultant Q vs V curve had an RMSE of 7.7 mV vs the measured NE curve.

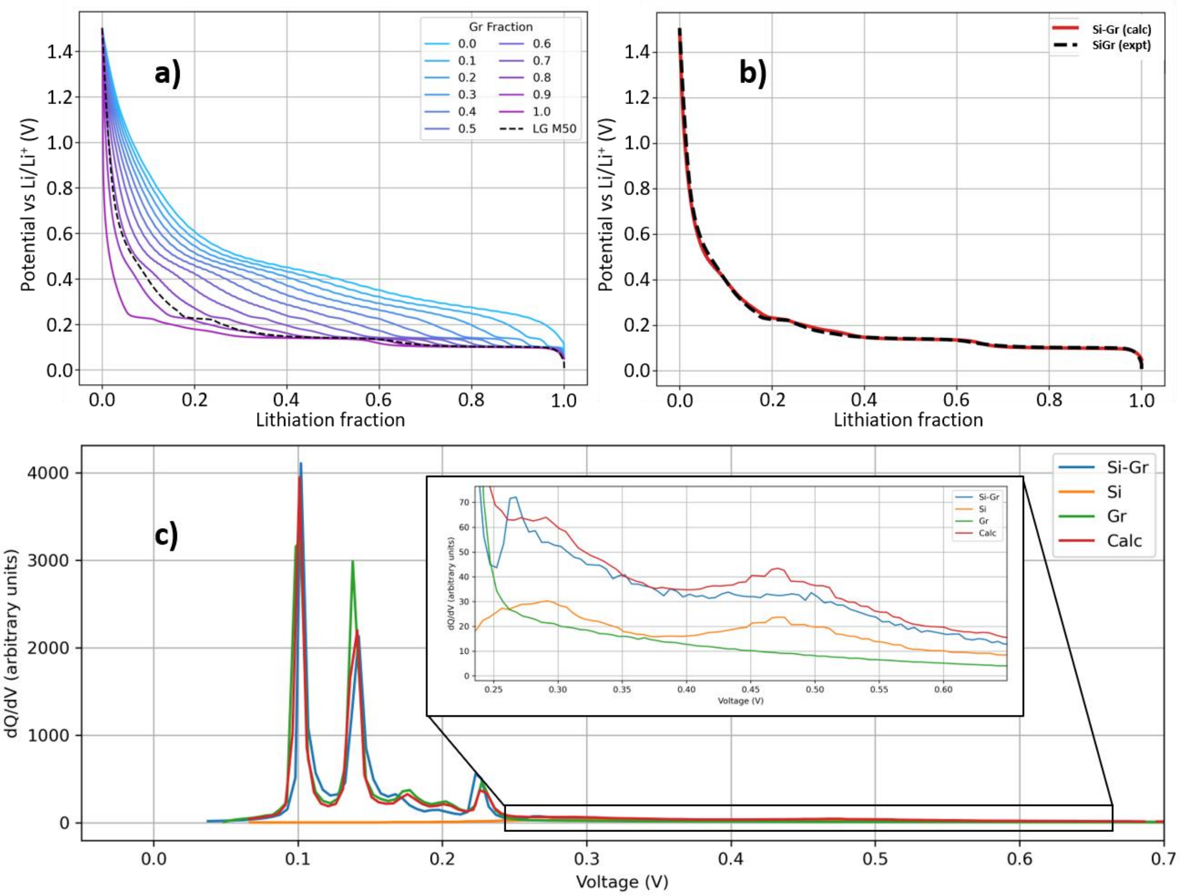

Figure S7. Reconstructing the S-Gr composite electrode voltage vs lithiation fraction (z) curve using the constituent Si and Gr datasets. a) Change in the V vs z profiles as a function of the Gr fraction present, from 0 (pure Si) to 1 (pure Gr). The measured Si-Gr voltage curve for an LG M50 cell is shown by the black dashed line. b) the resultant calculated curve from the fitting procedure (red) alongside the measured Si-Gr curve (blue). c) dQ/dV spectra for the measured Si-Gr, Si and Gr curves (blue, orange, and green, respectively), alongside the calculated Si-Gr curve from part b). inset: a zoomed region of the dQ/dV, showing the peaks corresponding to Si delithiation. All data shown are for delithiation processes (i.e. discharge on a full cell level).

The above results show that the V vs Q curve for the Si-Gr electrode can be accurately reproduced using a combination of the Si and Gr curves. However, we only have the half-cell V

vs Q curve for Si-Gr at BoL, so cannot use this method directly throughout the aging study. Instead, the methods of full cell OCV-fitting which have been detailed in other sources (such as [3]) can be used, but with minor adaptation. This involves introducing the new $\%Cap_{Gr}$ variable into the fitting procedure, as detailed in the main text. The method therefore contains five variables for the fitting procedure, the upper and lower lithiation fractions of both the PE and NE (x_{PE_lo}, x_{PE_up}, x_{NE_lo}, x_{NE_up}), and the fractional capacity of Gr in the NE ($\%Cap_{Gr}$).

The optimization minimizes the difference between the calculated and measured full cell voltage curves. The RMSE values corresponding to this difference were generally in the range of 3-15 mV, gradually increasing as the cells degrade and the measured 0.1C voltage drifted further from OCV values.

Results

For brevity, incremental capacity analysis (ICA, Figure S8 & S9) and degradation mode (DM, Figure S10-S13) analysis results were only shown for one cell per experimental condition in the main text. Below are the full results for all cells tested in this study. Alongside the DM analysis data are the extracted capacity data from the OCV-fitting procedure, showing the progression of each of the PE, NE, Gr and Si capacities (and offset) as a function of energy throughput during aging (Figure S10 & S12 for cells aged in the 0-30% and 0-100% SoC ranges, respectively).

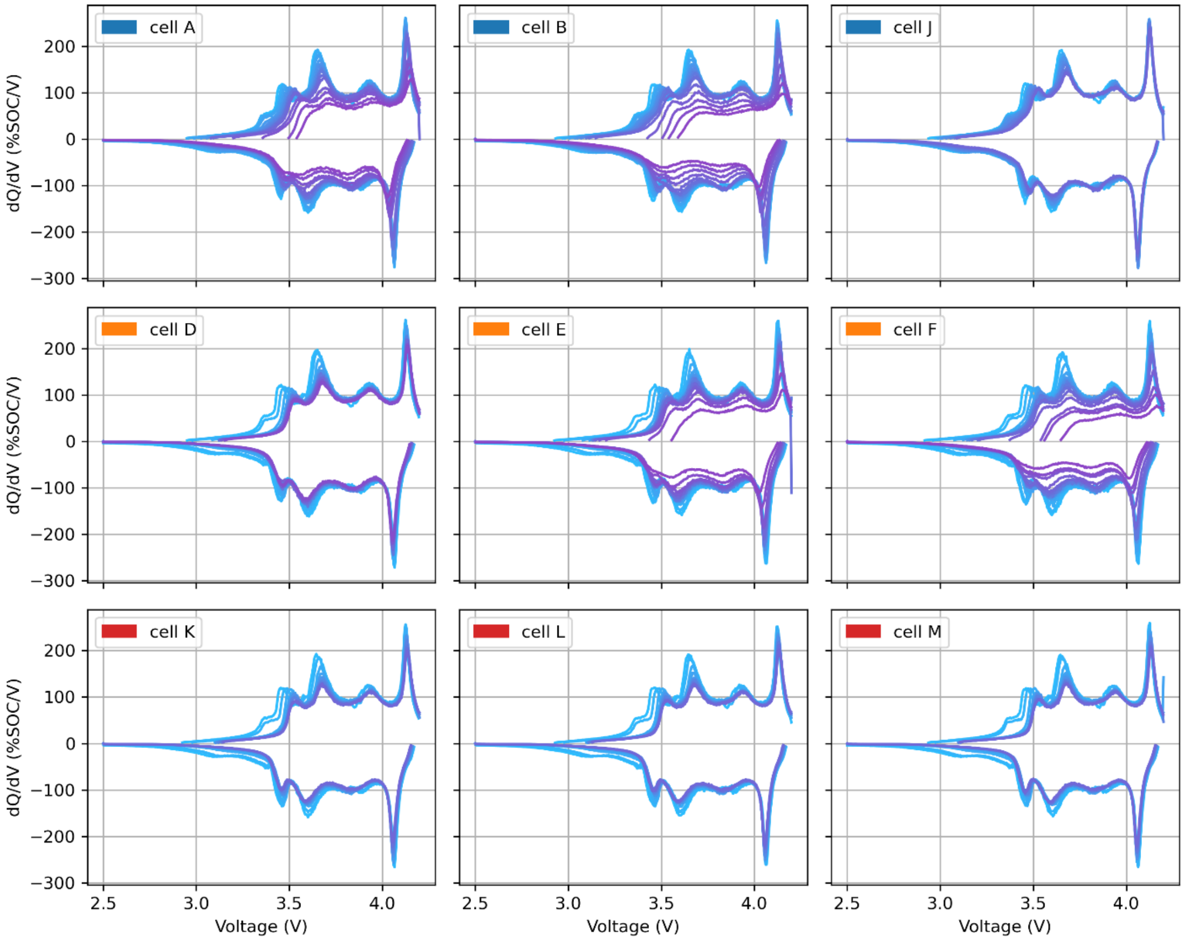

Figure S8. ICA plots for each cell cycled in the 0-30% SoC window, calculated from the 0.1C portion of the RPTs. Each plot corresponds to one cell being tested. The color of the plot legend corresponds to the temperature the cell was aged at (blue is 10°C, orange is 25°C, red is 40°C). The color of the line plot corresponds to the number of aging cycles from light blue (BoL) to dark purple (EoL), with approximately 77 full equivalent cycles between each.

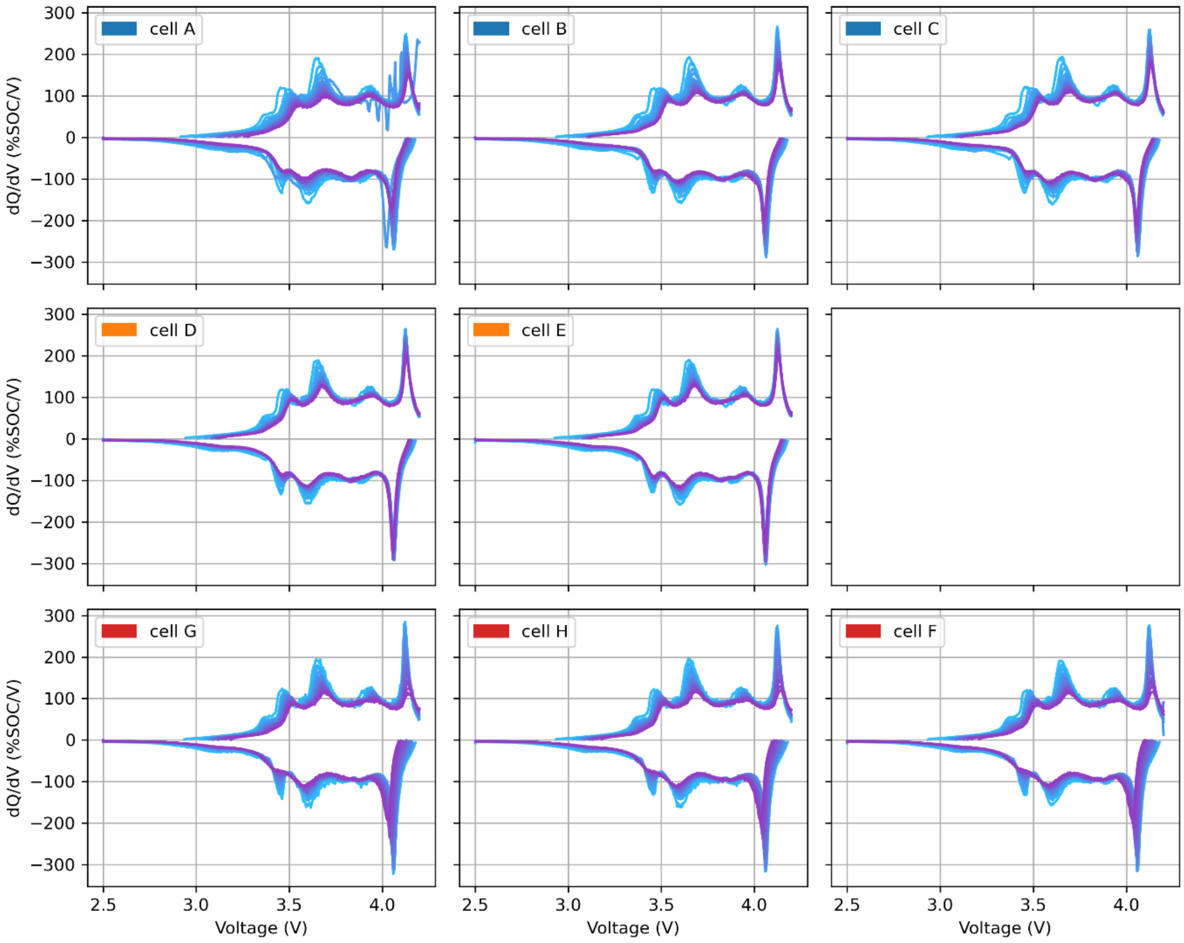

Figure S9. ICA plots for each cell cycled in the 0-100% SoC window, calculated from the 0.1C portion of the RPTs. Each plot corresponds to one cell being tested. The color of the plot legend corresponds to the temperature the cell was aged at (blue is 10°C, orange is 25°C, red is 40°C). The color of the line plot corresponds to the number of aging cycles from light blue (BoL) to dark purple (EoL), with approximately 77 full equivalent cycles between each. The noisy signal in one of the ICA profiles for cell A (top left) is due to a faulty electrical connection during measurement.

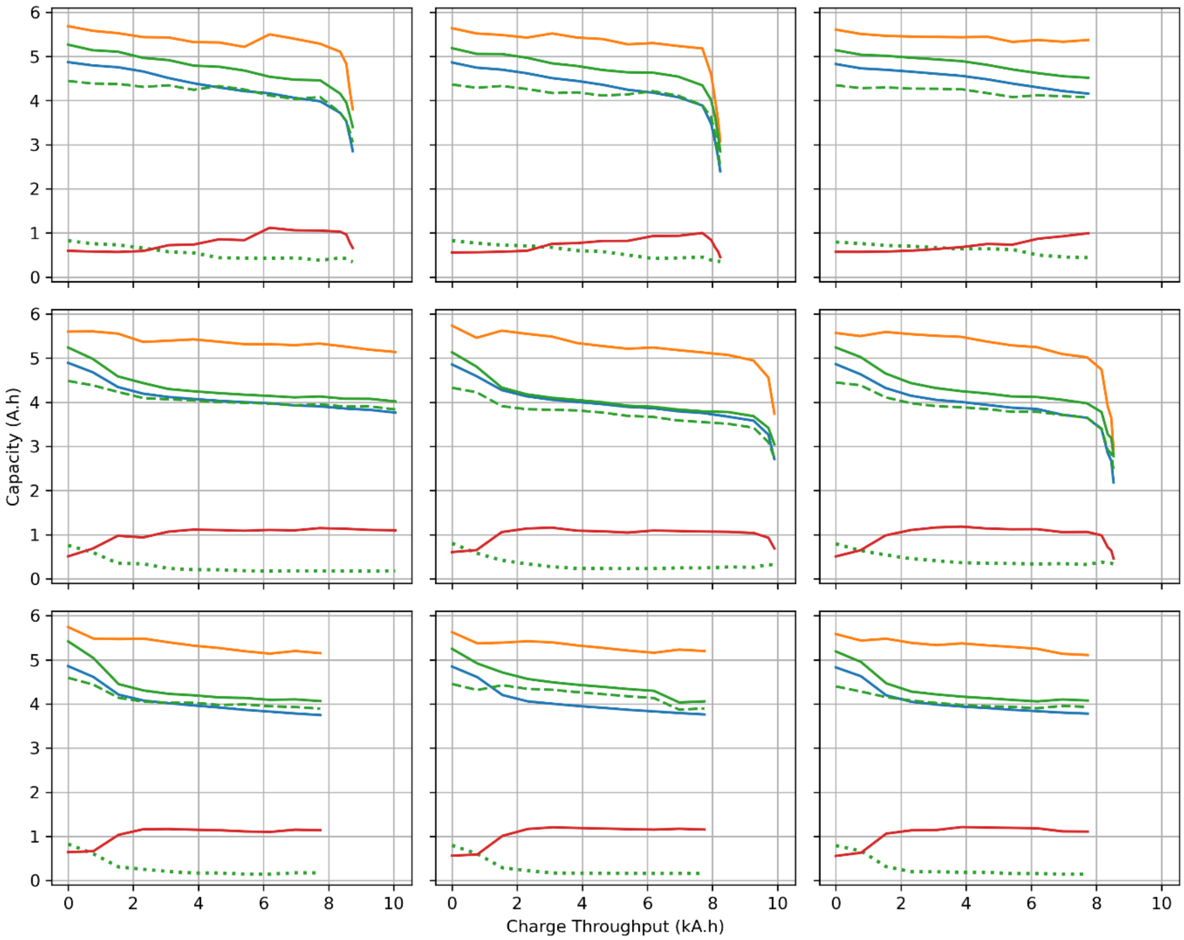

Figure S10. Capacity/offset data extracted from the OCV-fitting process for cells cycled in the 0-30% SoC window. This data is subsequently used for calculation of the Degradation Modes. Each plot corresponds to one cell being tested. Plots in the top row are cells aged at 10°C, the middle row are those aged at 25°C, and the bottom row are those aged at 40°C. Colors correspond to: cell capacity (blue), PE capacity (orange), NE capacity (green, solid), Gr capacity (green, dashed), Si capacity (green, dotted), electrode offset (red).

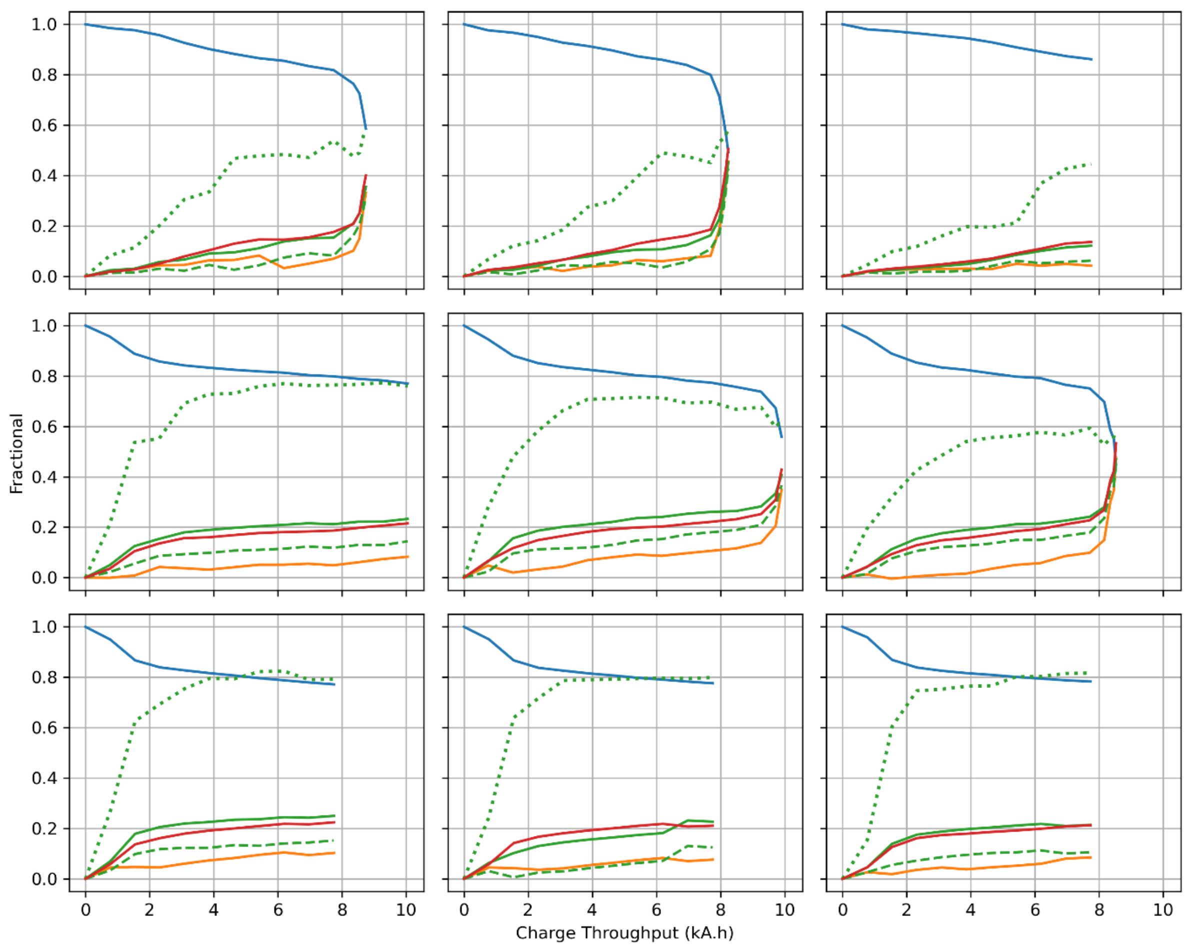

Figure S11. Degradation Mode Analysis data extracted from the OCV-fitting process for cells cycled in the 0-30% SoC window. Each plot corresponds to one cell being tested. Plots in the top row are cells aged at 10°C, the middle row are those aged at 25°C, and the bottom row are those aged at 40°C. Colors correspond to: cell SoH (blue), LAM-PE ((orange), LAM-NE (green, solid), LAM-NE-Gr (green, dashed), LAM-NE-Si (green, dotted), LLI (red). LLI is normalized by the BoL cell capacity.

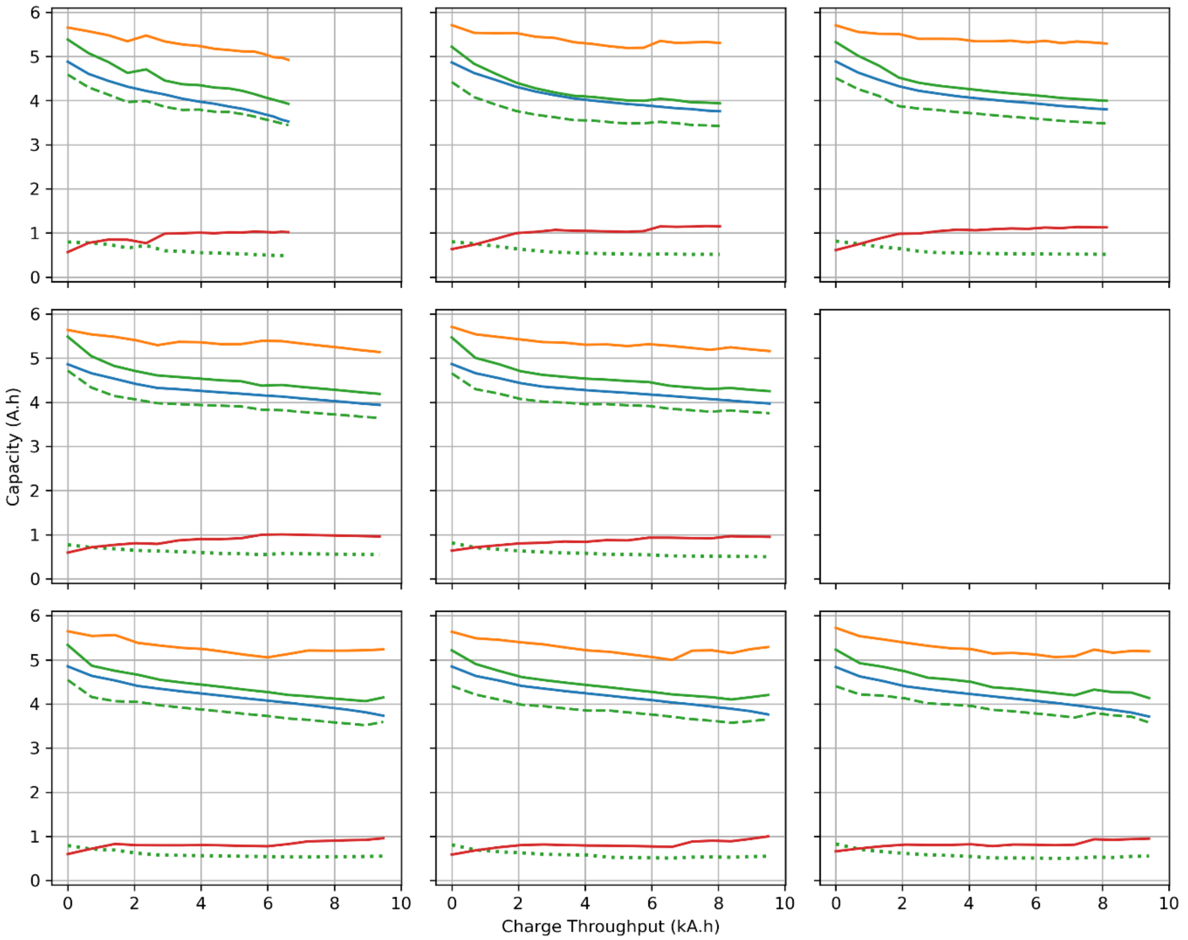

Figure S12. Capacity/offset data extracted from the OCV-fitting process for cells cycled in the 0-100% SoC window. This data is subsequently used for calculation of the Degradation Modes. Each plot corresponds to one cell being tested. Plots in the top row are cells aged at 10°C, the middle row are those aged at 25°C, and the bottom row are those aged at 40°C. Colors correspond to: cell capacity (blue), PE capacity (orange), NE capacity (green, solid), Gr capacity (green, dashed), Si capacity (green, dotted), electrode offset (red).

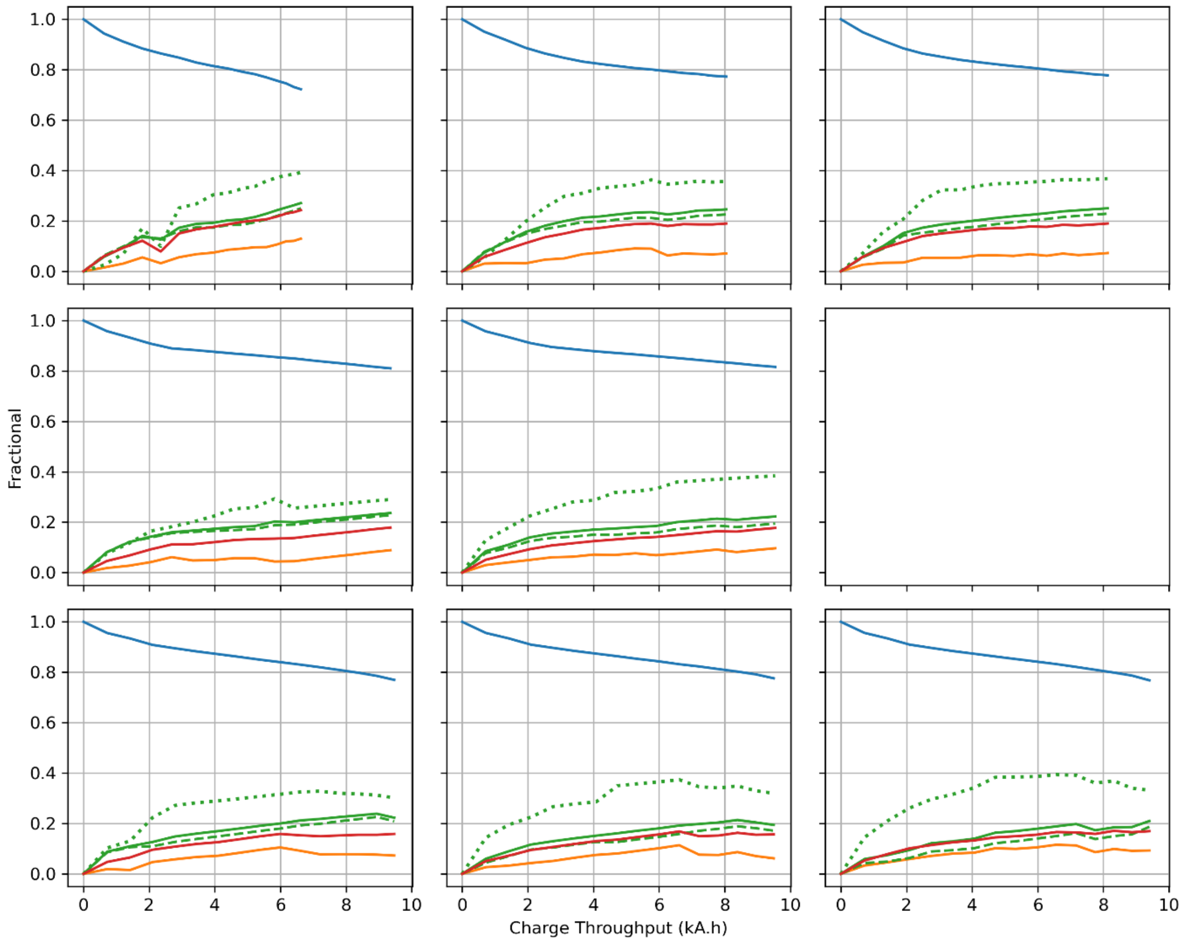

Figure S13. Degradation Mode Analysis data extracted from the OCV-fitting process for cells cycled in the 0-100% SoC window. Each plot corresponds to one cell being tested. Plots in the top row are cells aged at 10°C, the middle row are those aged at 25°C, and the bottom row are those aged at 40°C. Colors correspond to: cell SoH (blue), LAM-PE ((orange), LAM-NE (green, solid), LAM-NE-Gr (green, dashed), LAM-NE-Si (green, dotted), LLI (red). LLI is normalized by the BoL cell capacity.

References

(1) Chen, C.-H.; Planella, F. B.; O'Regan, K.; Gastol, D.; Widanage, W. D.; Kendrick, E. Development of Experimental Techniques for Parameterization of Multi-Scale Lithium-Ion Battery Models. *J. Electrochem. Soc.* **2020**, *167* (8), 80534. https://doi.org/10.1149/1945-

7111/ab9050.

(2) Kitada, K.; Pecher, O.; Magusin, P. C. M. M.; Groh, M. F.; Weatherup, R. S.; Grey, C. P. Unraveling the Reaction Mechanisms of SiO Anodes for Li-Ion Batteries by Combining in Situ 7Li and Ex Situ 7Li/29Si Solid-State NMR Spectroscopy. *J. Am. Chem. Soc.* **2019**, *141* (17), 7014–7027. https://doi.org/10.1021/jacs.9b01589.

(3) Prosser, R.; Offer, G.; Patel, Y. Lithium-Ion Diagnostics: The First Quantitative In-Operando Technique for Diagnosing Lithium Ion Battery Degradation Modes under Load with Realistic Thermal Boundary Conditions. *J. Electrochem. Soc.* **2021**, *168* (3), 030532. https://doi.org/10.1149/1945-7111/abed28.
